# Compensatory roles of Protein Related to DAN and Cerberus (PRDC) decrease in pulmonary arterial hypertension

**DOI:** 10.7150/ijbs.70247

**Published:** 2022-03-06

**Authors:** Ting He, Junzhi Zhang, Ting Qiao, Zhongjun Zhang, Hui Han, Chao Yang, Yong Chen, Yiwen Ruan, Liukun Meng

**Affiliations:** 1 Department of Anesthesiology, The Second Clinical Medical College, Jinan University (Shenzhen People's Hospital), Shenzhen 518020, China.; 2 Integrated Chinese and Western Medicine Postdoctoral Research Station, Jinan University, Guangzhou 510632, China.; 3 State Key Laboratory of Cardiovascular Disease, Fuwai Hospital, National Center for Cardiovascular Disease, Chinese Academy of Medical Sciences and Peking Union Medical College, Beijing 100037, China.; 4 Department of Anesthesiology, Shenzhen People's Hospital (The Second Clinical Medical College, Jinan University; The First Affiliated Hospital, Southern University of Science and Technology), Shenzhen 518020, China.; 5 Department of neurosurgery, Shenzhen University General Hospital, Shenzhen 518055, China.; 6 Department of neurosurgery, Shenzhen University Clinical Medical Academy, Shenzhen 518055, China.; 7 Department of Organ Transplantation and Thoracic Surgery, The First Affiliated Hospital of Guangzhou Medical University, Guangzhou 510120, China.

**Keywords:** Pulmonary arterial hypertension, pulmonary vascular remodeling, bone morphogenetic protein, monocrotaline, PRDC

## Abstract

Bone morphogenetic protein (BMP) signaling is commonly suppressed in patients with pulmonary arterial hypertension (PAH), but the compensatory mechanism of BMP signaling suppression is incompletely elucidated. This study aimed to investigate the role of PRDC, an antagonist of BMPs, in PAH and the underlying mechanism. Human lungs were collected and rat PAH was induced (monocrotaline, 60 mg/kg). BMP cascade and PRDC were detected in lungs and distal pulmonary artery smooth muscle cells (dPASMCs). *In vitro* cell experiments and *in vivo* supplementation of PRDC in hypertensive rats were subsequently performed. PRDC and BMP cascade all decreased in human and rat hypertensive lungs. Cell experiments confirmed that BMP2/4 inhibited dPASMCs proliferation by increasing cell cycle inhibitors (p21, p27), prevented dPASMCs migration by down-regulating MMP2/9 and up-regulating TIMP1/2 expression, and promoted dPASMCs apoptosis by up-regulating Bax, caspase3/9 and down-regulating Bcl-2 expression, as well as enhancing caspase3/7 activity, while, PRDC reversed the effects of BMP2/4 on dPASMCs proliferation, migration and apoptosis. *In vivo* trial found that PRDC supplementation deteriorated rat PAH in terms of pulmonary hemodynamics, vasculopathies and right ventricle hypertrophy. Taken together, compensatory decrease of PRDC in hypertensive lungs theoretically slow down the natural course of PAH, suggesting its therapeutic potential in PAH.

## Introduction

Pulmonary arterial hypertension (PAH) is a life-threatening disease due to progressive pulmonary arterial remodeling (PAR). As yet, the underling mechanism of PAR remains largely unelucidated, but burgeoning researches have confirmed that phenotype transformation of distal pulmonary artery smooth muscle cells (dPASMCs), including abnormal proliferation, migration, apoptotic resistance, plays central roles in PAR 1-2.

The major breakthrough in the mechanism research of PAH is the identification of BMP cascade dysfunction due to BMP receptor 2 (BMPR2) mutations or BMP cascade suppression in hypertensive lungs, which incurred differentiated dPASMCs reentry into a pro-proliferative and anti-apoptotic status 3-4. Subsequent studies found that heterozygous BMPR2 mutant mice exhibited PAR when exposed to hypoxia and BMPR2 targeted delivery to pulmonary vasculature ameliorated hypoxic PAH in rats 5-6. Further researches demonstrated that BMPs inhibited proliferation and induced apoptosis of dPASMCs *in vitro* and suppressed PAR 7-8. So, theoretically, proteins involved into BMP cascade are possible candidate targets in the mechanism research of PAH.

Previous studies found that PRDC, a potent BMP antagonist, showed stronger affinity and higher antagonistic ability to binding and blocking the actions of BMP than other antagonists 9. Co-treatment with PRDC antagonized the inhibitory effects of BMP on follicle-stimulating hormone stimulation of progesterone 10-11. In normal mice, PRDC was expressed in lungs during embryonic development 12. In addition, previous studies had found that PRDC was involved in the regulation of cell differentiation, proliferation and related to the cell phenotype transformation 13-14. Interestingly, dPASMCs proliferation, migration and apoptosis-resistance initialed and advanced PAR observed in PAH 1-2, thus, we speculate that PRDC plays important roles via BMP cascade in PAR and may be a potential therapeutic target for PAH.

This study aimed to explore the level of PRDC in hypertensive lungs, its roles in dPASMCs phenotype transformation and the effect of maintaining its plasma level on the extent of PAH.

## Materials and Methods

### Detection of PRDC in human lungs

Hypertensive lungs were collected from patients with idiopathic pulmonary arterial hypertension (IPAH, n=6) during heart-lung transplantation and control lungs (n=6) were obtained during lung resection surgeries for cancer at uninvolved regions distal to tumor margins. The clinical information, including etiology, status of BMPR2 mutation, medications at transplantation, New York Heart Association (NYHA) classification of cardiac function and pulmonary hemodynamic indices, were all supplemented (Table S1 and Table S2). Preliminary detection of PRDC was performed as the following segment “2.2.3 Immunofluorescence Staining”, “2.3.9 Western blot analysis” and “2.3.10 RT-PCR”.

Lung sample collection was approval by Research Ethics Committee of the first Affiliated Hospital of Guangzhou Medical University (Approval No. 2020-69) and the Research Ethics Committee of Fuwai Hospital, Chinese Academy of Medical Science & Peking Union Medical College (No. 2009-229). Written informed consent was obtained from all patients and this portion of the study conformed to the principles outlined in the Declaration of Helsinki.

### Rat PAH model induced by monocrotaline

#### Rat PAH model

As presented in Figure S1, Sprague-Dawley rats (Male, 6 weeks old, 200 to 240 g, Medical Experimental Animal Center of Guangdong Province) were randomly allocated into Control group (n=8), monocrotaline injection group (MCT-4W group, n=10), monocrotaline injection and saline administration group (MCT-Saline, n=10), and monocrotaline injection and PRDC administration group (MCT-PRDC, n=12). Rats in MCT-4W group were intraperitoneally injected monocrotaline (60 mg/kg, Sigma-Aldrich, St. Louis, Missouri, USA) and further kept for 4 weeks. Rats in MCT-Saline received saline administration via osmotic minipumps for 4 weeks after intraperitoneally monocrotaline injection (60 mg/kg). Rats in MCT-PRDC received PRDC administration via osmotic minipumps for 4 weeks after intraperitoneally monocrotaline injection (60 mg/kg).Rats in control group were intraperitoneally injected with isometric volume of isotonic saline solution and further kept for 4 weeks. Rats were kept in a room with 21 °C, relative humidity 50-70% under a normal light cycle. Right heart catheterization (RHC), sacrifice and tissue harvesting were performed at the 4^th^ week.

Osmotic minipumps (Model 2ML4; Alzet, CA, USA) were prepared according to the manufacturer′s instructions. After equilibrated in PBS at 37 °C for 48h, osmotic minipumps were filled with PRDC (15 μg) for MCT-PRDC group (n=12) and sterile 0.9% saline solution for MCT-Saline group (n=10) before subcutaneous implantation. SD Rats were anesthetized with isoflurane (1.5% vol/vol) and buprenorphine hydrochloride (0.03 mg/kg), and incisions were made in the dorsal and right cervical parts, then, minipump attached with a cannula prefilled with sterile heparin-saline were subcutaneously implanted and the cannula was tunnelled subcutaneously from dorsal region to cervical part and finally inserted into large ramifications of right external jugular vein.

Animal care was provided to all rats according to the ''Guide for the Care and Use of Laboratory Animals'' (National Institutes of Health publication no. 85-23, National Academy Press, Washington, DC, revised 2011). The animal care committee of Shenzhen People's Hospital approved this protocol (NO. LL-KT-201801009).

### Right heart catheterization and Tissue harvesting

Rats from Control group, MCT-4W group, MCT-Saline group and MCT-PRDC group received right heart catheterization (RHC) procedure at the4^th^ week for pulmonary hemodynamics as we routinely performed 7. Right ventricular systolic pressure (RVSP), pulmonary arterial systolic pressure (PASP) and mean pulmonary arterial pressure (mPAP) were recorded and the mean values were calculated. Immediately after RHC procedure, 4 °C sterile saline was perfused into pulmonary circulation to flush out the residual blood, after that, lungs were isolated and divided: parts immediately frozen in liquid nitrogen for further gene and protein analysis, and parts fixed in 10% neutral-buffered formalin, routinely processed for paraffin embedding and subsequent immunofluorescence staining. Right ventricular hypertrophy index (RVHI) was assessed by the ratio of right ventricle (RV) weight to that of left ventricle plus septum (LV+S), that is, RVHI=RV/(LV+S).The morphologic analysis in pulmonary vasculature were stained with hematoxylin-eosin or Weigert's elastic staining.

### Immunofluorescence Staining

As we previously reported 7, after antigen retrieval with microwave, lung sections of 4 μm thickness from human and rat lung tissues were immerged into blocking solution containing 0.3% Triton X-100 and 5% normal goat serum for 1 h at room temperature, and then sequentially be incubated with primary antibodies to PRDC (1:400 for human, 1:600 for rats, GeneTex, CA, USA), vWF (1:100, Abcam, Cambridge, UK) and α-SMA (1:100, Sigma-Aldrich, Saint Louis, USA) overnight at 4 °C. Next day, lung tissues were washed with PBS and incubated with secondary antibodies for 1 h at room temperature and then mounted with Roti-MountFluorCare DAPI (ZSGB-Bio, Beijing, China). Fluorescence was captured with the Leica SP8 confocal laser scanning microscope at 800-times magnification.

### Analysis of pulmonary vasculopathies

Our previous studies found that PAR in PAH mainly occurred in distal pulmonary arteries (dPAs) < 75 μm in external diameter (IED) 7. Lung sections stained with hematoxylin-eosin staining and the modified Weigert's method with Van Gieson's solution were used to document the grade of pulmonary vasculopathies in rats of Control group, MCT-4W, MCT-Saline and MCT-PRDC. Concretely, pulmonary vasculopathies in dPAs < 75 μm IED were demarcated as media hypertrophy (grade Ⅰ), medial hypertrophy with cellular intimal proliferation (grade Ⅱ), cellular intimal formation and stenosis (grade Ⅲ), according to the classification presented by Heath-Edwards 15. The extent of media hypertrophy was quantitatively analyzed by the percentage of medial wall thickness (MT%) in pulmonary arteries with well-defined internal and external elasticae; The degree of luminal stenosis for evident neointima formation was classified as: stenosis <50% and stenosis >50%. Evaluation of pulmonary vasculopathies in dPAs < 75 μm IED in each slide was performed by one investigator (H.Han) who was blinded to animal grouping and experimental protocol 7, 16.

### Wheat germ agglutinin staining

As we previously reported 7, 16, at the 4^th^ week, transversal myocardial tissues perpendicular to the ventricular septum from MCT-Saline and MCT-PRDC were routinely processed into paraffin-embedded specimens. Routine gross histologic examination on heart sections stained with hematoxylin and eosin. Quantification of myofiber diameters were performed by identifying cardiac myocyte membrane with FITC-conjugated wheat germ agglutinin (WGA, ab20528; Abcam). In each section, the cross-sectional area of 50 cardiomyocytes with circular-to-oval shape and nucleicentrally located were quantitatively analyzed at × 400 magnification with Image J software (National Institutes of Health, Bethesda, MD, USA).

### Cell experiments

#### Cell culture

As we previously reported 16, distal pulmonary arteries (dPAs) (greater than fourth generation) were dissected from lungs removed from healthy rats (8-wk-old male Sprague-Dawley rats). After denuding from adventitia and endothelium, dPASMCs were enzymatically digested from the tissue, harvested, and cultured in complete smooth muscle cell growth medium (SMCM, Cell Applications, INC.) containing 2% fetal bovine serum (FBS), 1% smooth muscle cell growth supplement, and 1% penicillin-streptomycin solution at 37 °C in a humidified atmosphere with 5% CO_2_-95% air. dPASMCs were subcultured when reached about 80-90% confluence, then, passages 4-6 generations were used for the following functional experiments. Briefly, dPASMCs were starved in resting SMCM (containing 0.2% FBS) for 24 h, then treated with recombinant PRDC (R&D Systems, Minneapolis, MN, USA) of indicated concentration for 1 h prior to stimulation with recombinant BMP2/4 (R&D Systems) for another 24 h 16.

### BrdU incorporation assay

dPASMCs proliferation was analyzed by measuring DNA synthesis with a colorimetric bromodeoxyuridine (BrdU) enzyme-linked immunosorbent assay kit (Roche, Roche Tech, Switzerland) according to the manufacturer′s instructions. Briefly, dPASMCs were cultured in 96-well plates and starved for 24 h prior to indicated treatments. BrdU labeling reagent was added into the medium incubation at 37 °C for 2 h. Then the labeling medium was replaced with FixDenat solution for incubation 30 min at 37 °C. Subsequently, FixDenat solution was removed and anti-BrdU-POD solution was added for incubation 90 min at room temperature, then, unconjugated antibody was removed by rinsing with wash solution. Finally, incorporation of BrdU absorbance values were detected at 370 nm (reference 492 nm) using a microplate reader (SPARK 10M, TECAN, Switzerland).

### Ki67 staining

dPASMCs were seeded on coverslips in 24-well plates for immunofluorescence assay. After starved for 24 h prior to indicated treatments, dPASMCs were fixed in 4% paraformaldehyde for 20 min and rinsed with PBS three times. Subsequently, dPASMCs were permeabilized with 0.3% Triton X-100 at room temperature for 10 min, and then blocked with 5% non-fat milk for 30 min. The primary antibody against ki67 (1:250, Abcam, Cambridge, UK) was incubated overnight at 4 °C. After washing three times with PBS, dPASMCs were incubated with Alexa Fluor 488-conjugated goat anti-rabbit IgG secondary antibody (1:500, Abcam) for 30 min at 37 °C in the dark, then, washed three times with PBS before being incubated with 4′,6-diamidino-2-phenylindole (DAPI) (1 µg/mL) (Sigma-Aldrich, St. Louis, Missouri, USA) for 5 min. Finally, images were captured under a fluorescence microscope (Leica DMi8, Wetzlar, Germany) and quantitative analysis was performed using Image J analysis software.

### TUNEL assay

Effects of PRDC on dPASMCs apoptosis were evaluated by terminal deoxynucleotidyl transferase-mediated dUTP nick end labeling (TUNEL) apoptosis detection kit according to the manufacturer's protocol (Roche, Roche Tech, Switzerland). Briefly, cells were seeded on coverslips in 24-wellplates at a density of 2×10^5^ cells/well. After starved for 24 h prior to indicated treatments, cell slides were fixed in 4% paraformaldehyde for 60 min, washed with PBS, permeabilized using 0.1% Triton X-100 for 2 min on ice and then stained with TUNEL detecting liquid for 1 h at room temperature in dark. Subsequently, cells were washed twice with PBS. DAPI was used to stain the cell nuclei for 10 min at room temperature. Fluorescent signals were then visualized by fluorescence microscope (Leica DMi8) and the percentage of TUNEL-positive cells was assessed in five randomly selected microscopic fields in each cell slide.

### Annexin V-FITC/PI apoptosis assay

To further quantify the number of apoptotic dPASMCs, Annexin V-FITC/PI apoptosis detection assay (BD Biosciences, New Jersey, USA) was performed according to the manufacturer's instructions. At the end of the treatment period, 1×10^6^ cells/ml were collected, washed twice in cold PBS and resuspended in 500 μl binding buffer. Subsequently, 5 μl Annexin V-FITC and 5 μl PI were added to each sample, gently vortexed and incubated in the dark for 15 min at room temperature. Then the fluorescence of the stained dPASMCs was immediately detected by a flow cytometer (BD Biosciences) and analyzed using Cell Quest Pro software.

### Caspase activity assay

Caspase3/7 activities were measured using the Caspase-Glo® 3/7 Assay (Promega, Madison, WI, USA) according to the manufacturer's protocol. Briefly, dPASMCs were lysed, 50 μg total proteins were added to the reaction buffer containing Ac-DEVD-pNA (2 mM), and then incubated at 37 °C for 2 h. The absorbance of pNA cleavaged from its corresponding precursors was measured using a microplate reader (SPARK 10M, TECAN, Switzerland) at 405 nm.

### Would healing assay

dPASMCs migration was determined by wound-healing assay as described in previous studies. dPASMCs were seeded in 6-well plates and grown to 90-95% confluence. Then the confluent cell monolayer was scratched to generate a cell-free gap using pipette tip. dPASMCs were washed with PBS twice to remove debris and replenished with resting SMCM. dPASMCs migration towards to the midline of the scratch was captured at 0 h and16 h in the same area with a light microscope (CKX31, Olympus, Tokyo, Japan).

### Transwell migration analysis

Transwell migration analysis was performed using transwell chambers (Corning, 24-well, 8 μm pore-size; NY, USA) 16. dPASMCs were plated into the upper chamber in resting SMCM (containing different concentration of PRDC and BMP2/4), then, complete SMCM was added into the lower chamber as the chemoattractant. After incubation at 37 °C for 16 h, migrated cells attached to the bottom surface of the insert membrane were fixed with methanol for 30 min and further stained with 0.1% crystal violet (Sigma-Aldrich) for 30 min at room temperature. Residual nonmigrated cells on the upper surface of the insert membrane were gently wiped off with a cotton swab. Quantification was performed by counting the number of cells in six randomly selected fields under the light microscope (CKX31, Olympus).

### Western blot analysis

Total proteins were extracted from lungs, dPASMCs by using RIPA lysis buffer containing phenylmethanesulfonyl fluoride (PMSF) on ice for at least 30 min. Lysates were centrifuged at 4 °C and 12,000 rpm for 15 min, then, supernatants were collected for the determination of protein concentrations using the BCA protein assay kit (Beyotime Biotechnology, Guangzhou, China). The equal amounts of protein were loaded and separated by 10% or 12% sodium dodecyl sulfate-polyacrylamide gel electrophoresis (SDS-PAGE) and transferred onto PDVF membranes (Millipore, Billerica, MA,USA, pore size of 0.22 µm). Then, the membranes were blocked in 5% non-fat dry milk for 2 h at room temperature, followed by incubation overnight at 4℃ with appropriate primary antibodies including cyclin D1 (1:10000, Abcam), proliferating cell nuclear antigen (PCNA), Id-1 (1 μg/ml, Abcam), p27 (1:5000, Abcam), MMP2, MMP9, TIMP1, TIMP2, Caspase9, Bcl-2, BMPR2, PRDC (1:1000, Abcam), Total-Smad1/5/8 (T-Smad1/5/8) (1:500, Abcam), p21 (1 μg/ml, Invitrogen, Carlsbad, CA, USA), Caspase3, Bax, phospho-Smad1/5/8 (p-Smad1/5/8), GAPDH (1:1000, Cell Signaling Technology, MA, USA), respectively. After washing three times with TBST, the membranes were incubated with either anti-mouse or anti-rabbit IgG HRP-conjugated (1:1000, Beyotime Biotechnology, Guangzhou, China) corresponding secondary antibodies at room temperature for 1 h. Subsequently, the protein band intensities were visualized using the enhanced chemiluminescence detection reagents (ECL, Millipore, USA). GAPDH served as an endogenous control to normalize the expression level of detected proteins. The relative intensities of the proteins were quantified using software Quantity One.

### RT-PCR

Total RNA from lungs or dPASMCs, was extracted using the TRIzol reagent (Invitrogen) and reversely transcribed into cDNA using an AMV reverse transcriptase kit (Promega, Madison, WI, USA) following the manufacturer's instructions. PRDC, BMPR2 and Id-1 RNA levels were determined by RT-PCR using SYBR Green PCR Master Mix (Qiagen, DU, Germany) and StepOnePlus Real-Time PCR System (Thermo fisher Scientific, Waltham, MA, USA). The expression of PRDC, BMPR2 and Id-1 were normalized against GAPDH and relatively fold change was quantified using 2^-ΔΔCt^ method. The primer sequences used for RT-PCR were presented in Table S3.

### Statistical Analysis

All experiments were independently performed in triplicate. Data were expressed as the mean ± standard error of mean, and statistical analyses were carried out using GraphPad Prism 8.0 (GraphPad Software, Inc, SanDiego, CA). Statistical differences between two independent groups were determined using student's t-test. Multiple group comparisons were performed by one-way ANOVA followed by Tukey's or Dunnett's T3 test. A value of *p*<0.05 was considered to be statistically significant difference.

## Results

### PRDC level significantly decreased in lungs of PAH patients

**Figure 1:**
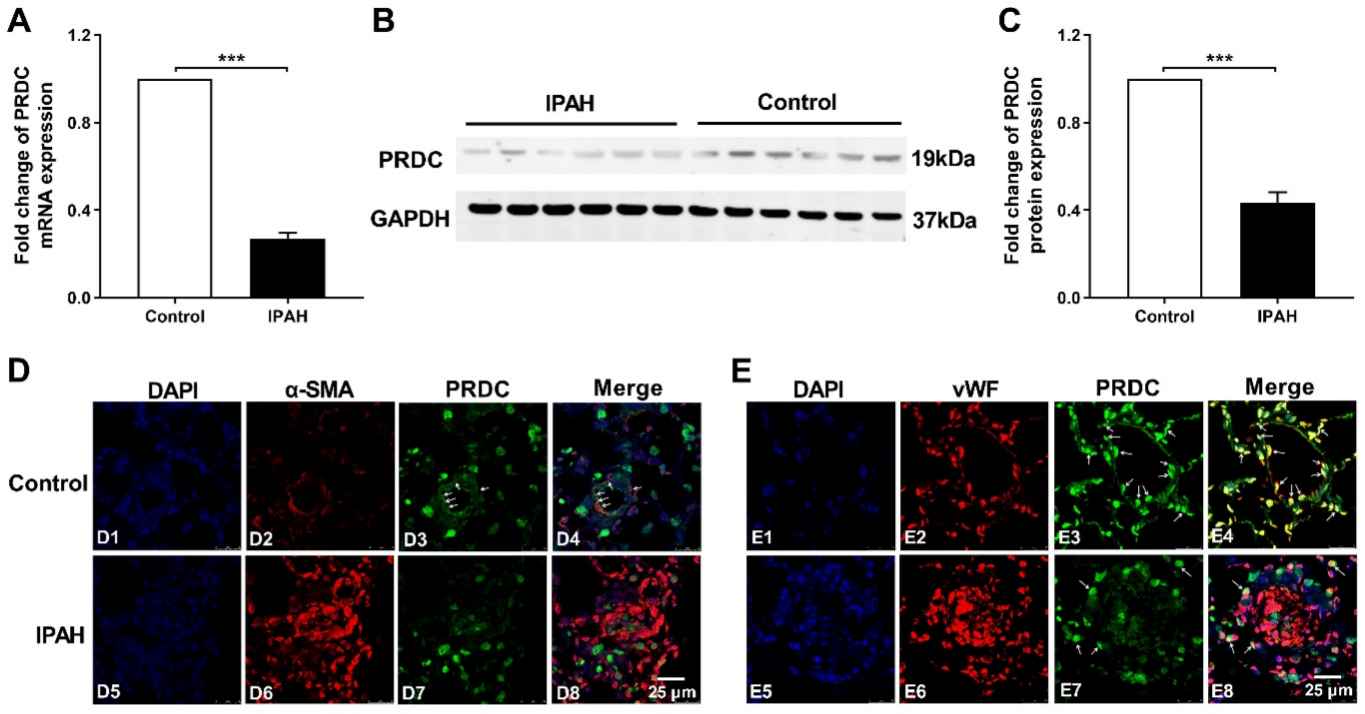
** PRDC level in human hypertensive lungs with IPAH. (A)** mRNA level of PRDC expression in human lungs (n=6). **(B)** Representative image of western blot analyses in human lungs. **(C)** Densiometric analysis of PRDC normalized to GAPDH in human lungs (n=6).** (D)** Immunofluorescence for PRDC (green), smooth muscle α-actin (α-SMA, red) and nuclei (blue) in normal pulmonary arteries of control lungs (D1-D4) and remodeled pulmonary arteries of lungs with IPAH (D5-D8) (Scale bar, 25 µm). White arrows indicate colocalization of PRDC and α-SMA. **(E)** Immunofluorescence for PRDC (green), von Willebrand factor (vWF; red) and nuclei (blue) in normal pulmonary arteries of control lungs (E1-E4) and remodeled pulmonary arteries of lungs with IPAH (E5-E8) (Scale bar, 25 µm). White arrows indicate the colocalization of PRDC and vWF. ***P < 0.001.

As demonstrated by Figure 1A-C, lung PRDC expression, determined by RT-PCR and Western blotting analysis, significantly decreased in patients with IPAH as compared with those in control subjects. Immunofluorescent staining found that strong intensity staining of PRDC, colocalized with smooth muscle α-actin (a smooth muscle marker) and von Willebrand factor (an endothelium biomarker), presented in normal pulmonary arteries of control human lungs (Figure 1D4, E4), but slight staining of PRDC in severely remodeled and obliterated pulmonary arteries of lungs from patients with IPAH (Figure 1D8, E8).

### Lung PRDC level decreased in PAH model in rats

As presented in Figure S2 A-D, values for RVSP, PASP, mPAP and RVHI in rats of MCT-4W increased to a higher level as compared with those of control group. As showed in Figure S2 E2, extensive muscularization and slight intimal hyperplasia were incurred in pulmonary arteries in MCT-4W. Apparently, monocrotaline injection for 4 weeks successfully induced a hypertensive status in rat lungs.

**Figure 2:**
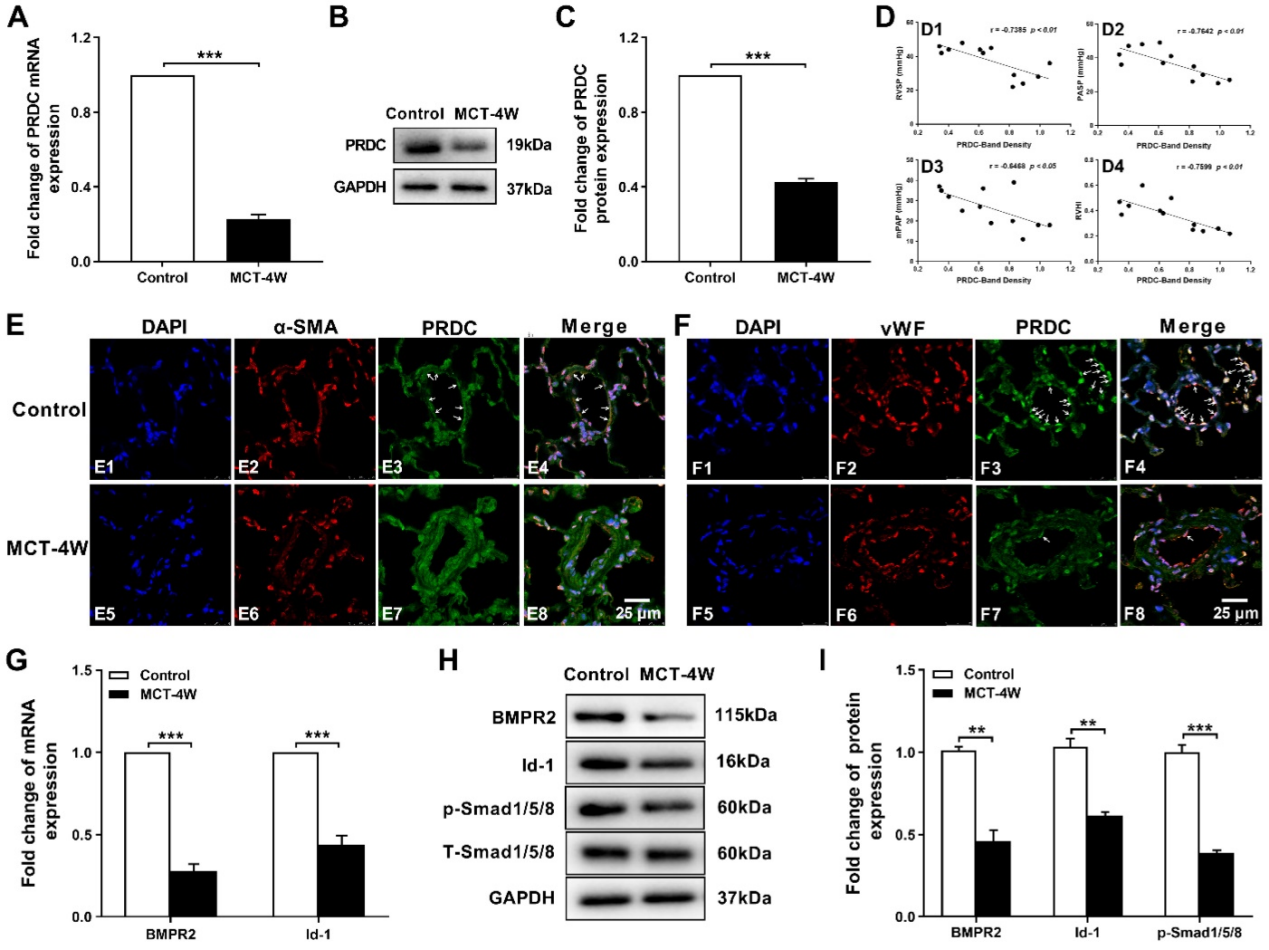
** PRDC and BMP signal in rat hypertensive lungs. (A-C)** PRDC expression in rat lungs: mRNA level of PRDC (A), representative immunoblotting image of PRDC (B) and pooled data of densitometric analysis of PRDC (C) (n=3-5). **(D)** Correlation between lung PRDC band density and RVSP (D1), PASP (D2), mPAP (D3) and RVHI (D4). **(E)** Immunofluorescence for PRDC (green), smooth muscle α-actin (α-SMA, red) and nuclei (blue) in normal pulmonary arteries and remodeled pulmonary arteries of rat lungs (Scale bar, 25 µm). White arrows indicate colocalization of PRDC and α-SMA. **(F)** Immunofluorescence for PRDC (green), von Willebrand factor (vWF; red) and nuclei (blue) in normal pulmonary arteries and remodeled pulmonary arteries of rat lungs (Scale bar, 25 µm). White arrows indicate the colocalization of PRDC and vWF. **(G-I)** Expression pattern of BMP cascade in lungs with monocrotaline induced PAH: mRNA level of BMPR2 and Id-1 (G); Representative immunoblotting image of BMP cascade (H) and pooled data of densitometric analysis (I) (n=3-5). **P < 0.01, ***P < 0.001,*^NS^ p* > 0.05.

Subsequent RT-PCR and Western-blotting analysis demonstrated a significant decrease of PRDC level in rat lungs from MCT-4W as compared with that of control group (Figure 2A-C). Then, as demonstrated in Figure 2D, after the densities of the PRDC bands on Western blotting are normalized to the density of the corresponding GAPDH band, further linear regression analysis revealed that lung PRDC protein level negatively correlated with the severity of MCT-PAH as measured by RVSP, PASP, mPAP and RVHI. Immunofluorescence staining for PRDC detected intense expression of PRDC in lungs of control group and predominantly confined to the medial layer (Figure 2E4) and endothelial layer of normal pulmonary arterioles (Figure 2F4), but slight expression of PRDC was observed in the medial layer (Figure 2E8) and endothelial layer of remodeled pulmonary arterioles of MCT-4W (Figure 2F8), while intense expression of PRDC was detected in the adventitia both in control group (Figure 2E4, F4) and MCT-4W group (Figure 2E8, F8), indicating PAH induced by MCT significantly suppressed the PRDC expression in the endothelial and medial layer, but had no effect on the adventitia of the remodeled pulmonary arterioles.

As presented in Figure 2G-I, when compared with those of control group, the mRNA and protein level of BMPR2, inhibitor of DNA binding protein-1 (Id-1) and the phosphorylation level of drosophila mothers against decapentaplegic protein 1/5/8 (p-Smad1/5/8) were all significantly down-regulated in hypertensive lungs induced by monocrotaline injection, implicating BMP cascade was comprehensively inhibited.

### PRDC promoted the transform of dPASMCs phenotype

#### Expression of PRDC in dPASMCs

dPASMCs cultured in resting SMCM for 48 h were mostly synchronized into G0/G1 phase, but after re-stimulation with complete SMCM for 12 h or 24 h, dPASMCs in S and G2/M phase increased but dPASMCs in G0/G1 phase decreased to a comparable level (Figure S3A-B). After stimulation with complete SMCM for 12 h or 24 h, the mRNA expression and the amount of PRDC secreted into the cell culture medium significantly decreased comparing with those of dPASMCs at 0 h (Figure S3C-D).

#### PRDC promoted dPASMCs proliferation

**Figure 3:**
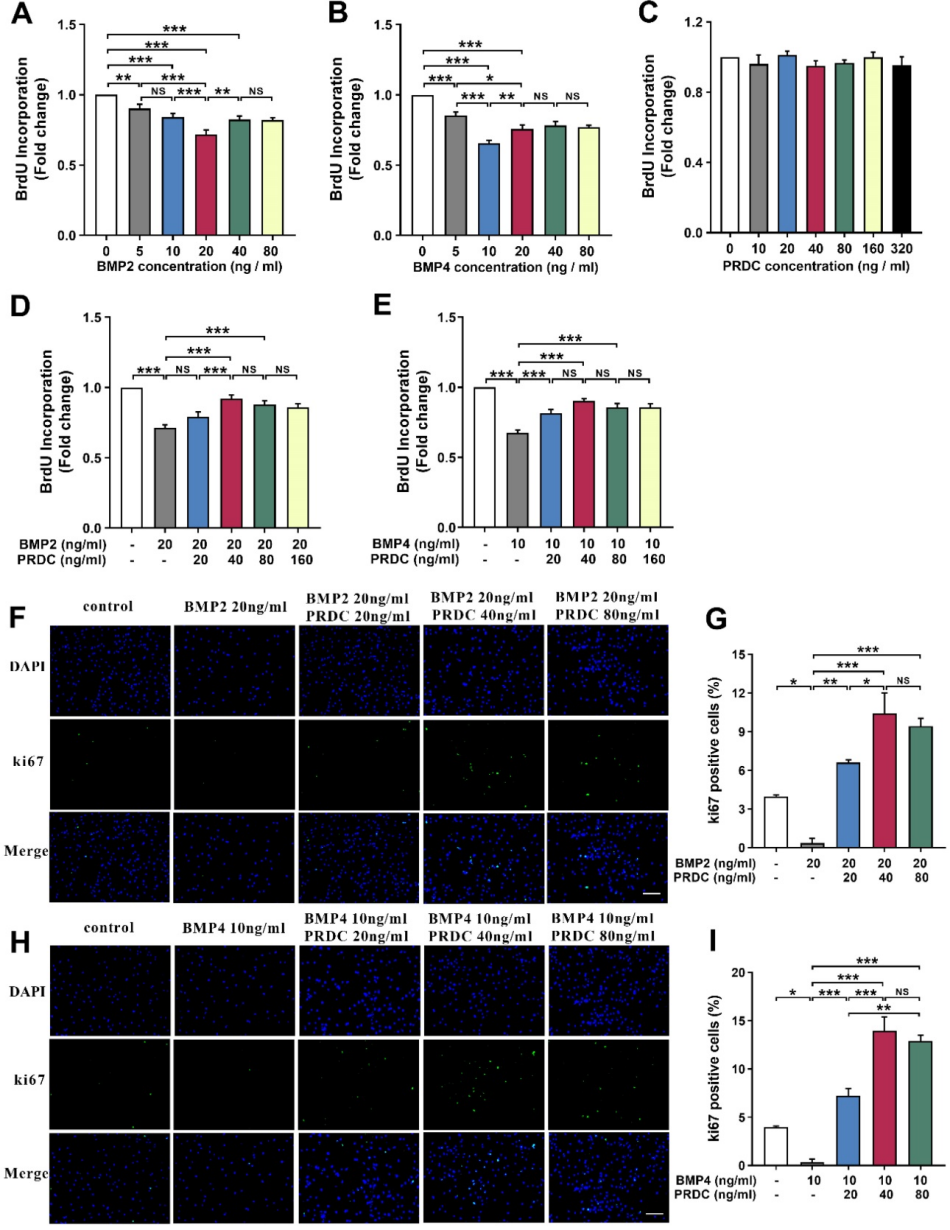
** PRDC reversed the anti-proliferation effects of BMP2/4 on dPASMCs. (A-B)** BMP2/4 suppressed dPASMCs proliferation (n=12). **(C)** Direct roles of PRDC in dPASMCs proliferation (n=3). **(D-E)** PRDC antagonized the inhibitory effects of BMP2/4 on dPASMCs proliferation (n=12). **(F-G)** Representative immunofluorescence staining (F) and quantitative analysis (G) for Ki-67 in dPASMCs exposed to BMP2 or BMP2+PRDC (Scale bar, 50 µm) (n=4). **(H-I)** Representative immunofluorescence staining (H) and quantitative analysis (I) for Ki-67 in dPASMCs exposed to BMP4 or BMP4+PRDC (Scale bar, 50 µm) (n=4). *P < 0.05, **P < 0.01, ***P < 0.001, ^NS^ p > 0.05.

BrdU incorporation assay demonstrated that BMP2/4 suppressed dPASMCs proliferation in a dose-dependent way and exerted a better inhibitory effect at 20 ng/ml and 10 ng/ml, respectively (Figure 3A-B). However, no direct effects of PRDC alone were observed on the proliferation of dPASMCs (Figure 3C). As showed in Figure 3D-E, the proliferation of dPASMCs restrained by BMP2 (20 ng/ml) or BMP4 (10 ng/ml) was regained by PRDC in a concentration-dependent way and almost recovered at 40 ng/ml.

Immunofluorescence staining for Ki67, a marker for cell proliferation, demonstrated that the percentage of Ki67-positive cells in dPASMCs exposed to BMP2 (20 ng/ml) or BMP4 (10 ng/ml) was much lower than that of dPASMCs in control group, while, PRDC (20, 40, 80 ng/ml) could substantially and dose-dependently reverse this trend incurred by BMP2 (20 ng/ml) or BMP4 (10 ng/ml) (Figure 3F-I).

**Figure 4:**
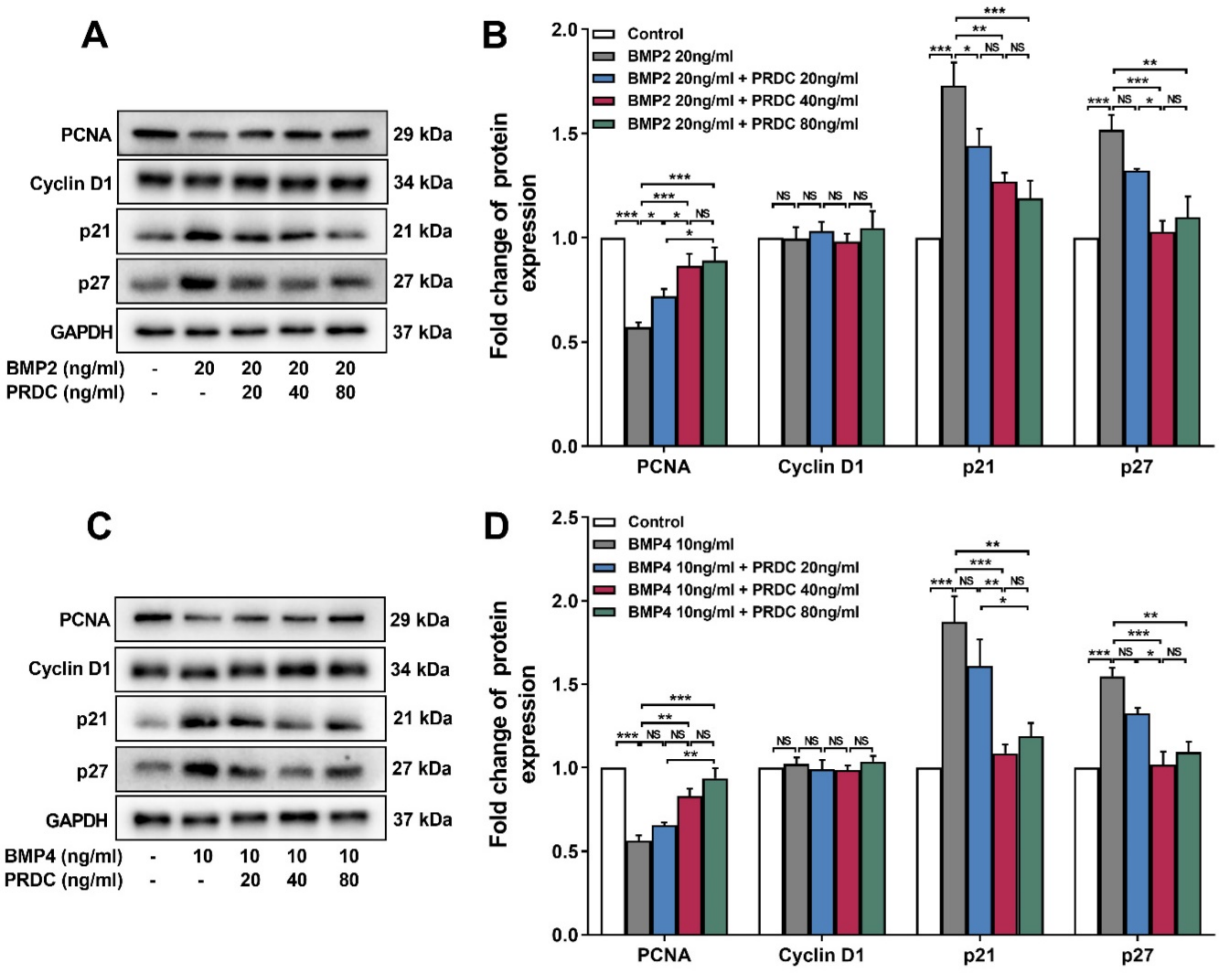
** Expression changes of proliferating cell nuclear antigen and cell cycle cytokines. (A)** Representative image of western blot analyses in dPASMCs exposed to BMP2 or BMP2+PRDC. **(B)** Densiometric analysis of PCNA, CyclinD1, p21 and p27 normalized to GAPDH in dPASMCs exposed to BMP2 or BMP2+PRDC (n=3-4). **(C)** Representative image of western blot analyses in dPASMCs exposed to BMP4 or BMP4+PRDC. **(D)** Densiometric analysis of PCNA, Cyclin D1, p21 and p27 in dPASMCs exposed to BMP4 or BMP4+PRDC (n=3). *P < 0.05, **P < 0.01, ***P <0.001,*^NS^ p* > 0.05.

Western blot analysis showed that BMP2 (20 ng/ml) or BMP4 (10 ng/ml) significantly down-regulated the level of proliferating cell nuclear antigen (PCNA, an indicator of cell proliferation), up-regulated p21 and p27 level, but had no effect on cyclin D1 expression as compared with control group, but pre-treatment with PRDC (20, 40, 80 ng/ml) prevented the down-regulation of PCNA and up-regulation of p21, p27 induced by BMP2 (20 ng/ml) or BMP4 (10 ng/ml) (Figure 4A-D), indicating that regulatory cyclin-dependent kinase inhibitors (p21 and p27) partially responsible for the observed blocking effect of PRDC on BMP2/4 axis on dPASMCs proliferation.

#### PRDC accelerated dPASMCs migration

**Figure 5:**
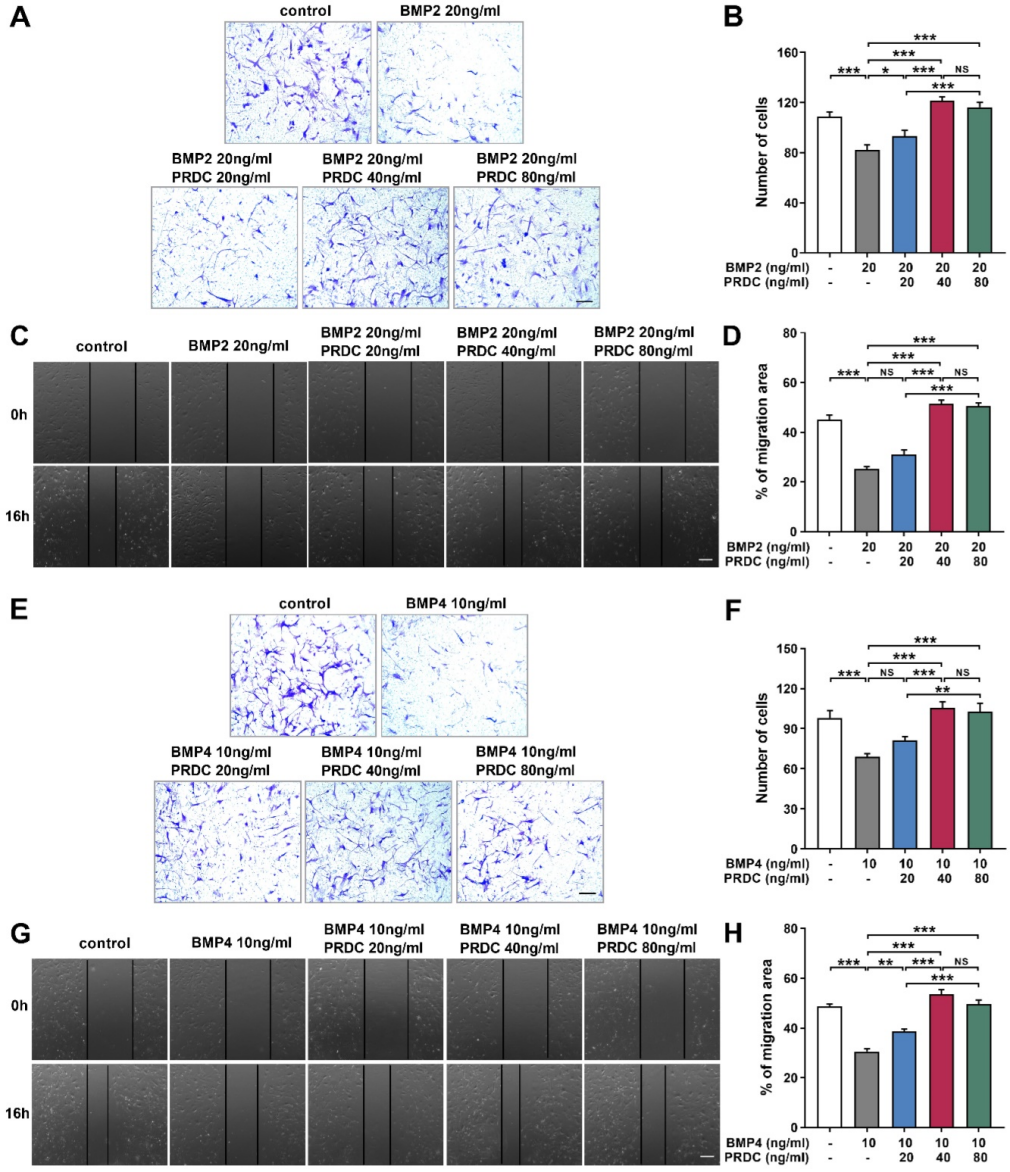
** PRDC partially abolished anti-migration induced by BMP2/4 in dPASMCs. (A)** Representative images of dPASMCs migrated and attached to the membrane bottom surface of transwell chamber exposed to BMP2 or BMP2+PRDC, Scale bar, 50 µm. **(B)** Number of migrated dPASMCs exposed to BMP2 or BMP2+PRDC, which was averaged from six randomly selected fields (n=6). **(C)** Representative images of wound-healing assay in dPASMCs exposed to BMP2 or BMP2+PRDC. **(D)** Quantitative analysis of migration area of dPASMCs exposed to BMP2 or BMP2+PRDC (n=6). **(E)** Representative images of dPASMCs migrated and attached to the membrane bottom surface of transwell chamber exposed to BMP4 or BMP4+PRDC, Scale bar, 50 µm. **(F)** Number of migrated dPASMCs exposed to BMP4 or BMP4+PRDC, which was averaged from six randomly selected fields (n=6). **(G)** Representative images of wound-healing assay in dPASMCs exposed to BMP4 or BMP4+PRDC. **(H)** Quantitative analysis of migration area of dPASMCs exposed to BMP4 or BMP4+PRDC (n=6). *P < 0.05, **P < 0.01, ***P < 0.001,*^NS^ p* >0.05.

As demonstrated in Figure 5A-B, E-F, in transwell assay, the number of migrated dPASMCs apparently decreased to a much lower value when subjected to BMP2 (20 ng/ml) or BMP4 (10 ng/ml) as compared with control group. Interestingly, PRDC (20, 40, 80 ng/ml) could substantially reverse the suppression of BMP2 (20 ng/ml) or BMP4 (10 ng/ml) on dPASMCs migration. Furthermore, in the wound-healing assay, BMP2 (20 ng/ml) or BMP4 (10 ng/ml) treatment induced retard of scratch closure was blocked by PRDC (20, 40, 80 ng/ml), further validating the blocking effect of PRDC on BMP2/4 axis on dPASMCs migration (Figure 5C-D, G-H).

**Figure 6:**
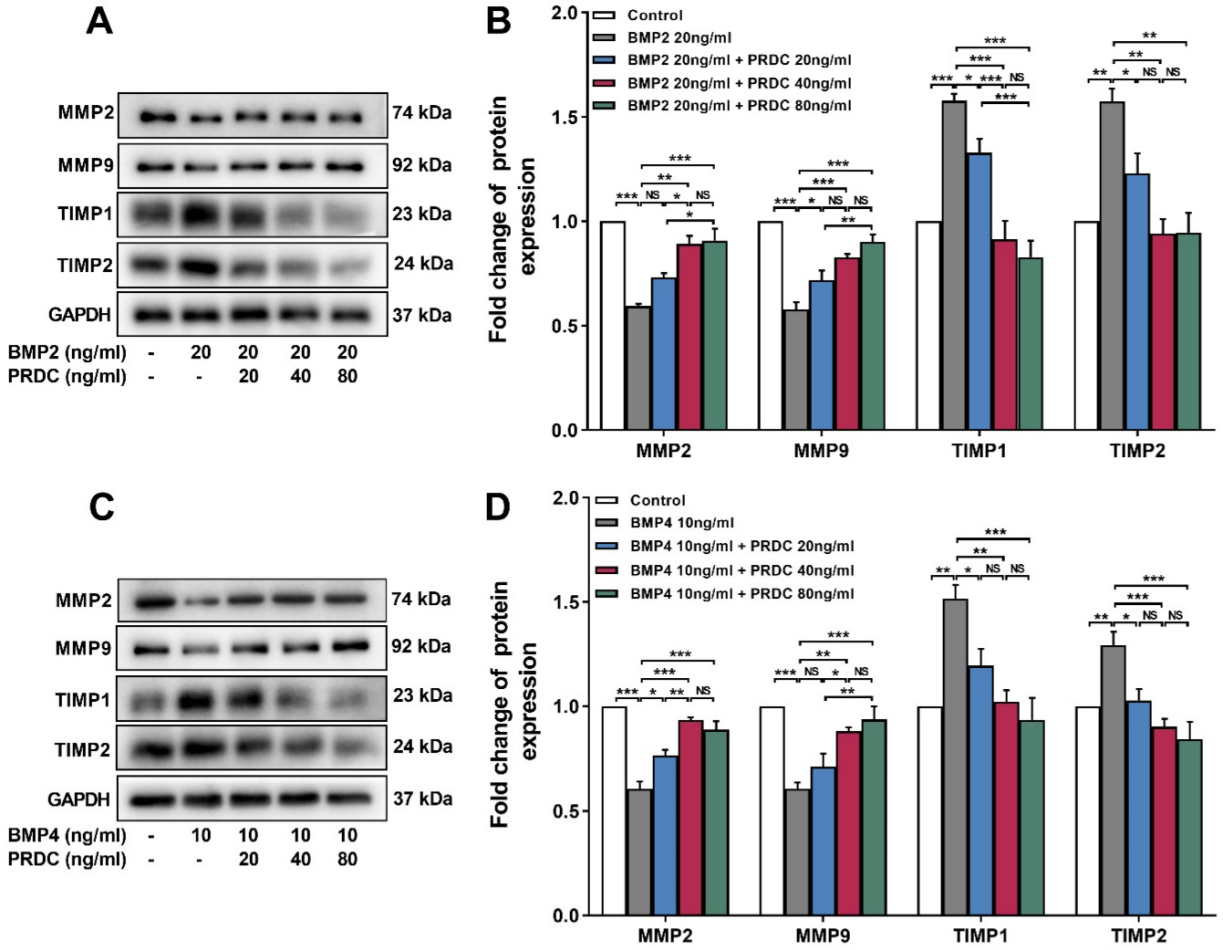
** Expression changes of MMPs/TIMPs in dPASMCs. (A)** Western blotting images of MMP2, MMP9, TIMP1 and TIMP2 in dPASMCs exposed to BMP2 or BMP2+PRDC. **(B)** Densiometric quantification of MMP2, MMP9, TIMP1and TIMP2 in dPASMCs exposed to BMP2 or BMP2+PRDC (n=3). **(C)** Western blotting images of MMP2, MMP9, TIMP1 and TIMP2 in dPASMCs exposed to BMP4 or BMP4+PRDC. **(D)** Densiometric quantification of MMP2, MMP9, TIMP1 and TIMP2 in dPASMCs exposed to BMP4 or BMP4+PRDC (n=3-4). *P < 0.05, **P < 0.01, ***P < 0.001,*^NS^ p* > 0.05.

Moreover, Western blot assay showed that cell migration-related proteins MMP2, MMP9 expression displayed a significant decrease but TIMP1, TIMP2 expression demonstrated a significant increase in response to BMP2 (20 ng/ml) or BMP4 (10 ng/ml) stimulation, but those changes in MMPs/TIMPs system were reversed by co-treatment with PRDC (20, 40, 80 ng/ml) (Figure 6A-D).

#### PRDC inhibited dPASMCs apoptosis

**Figure 7:**
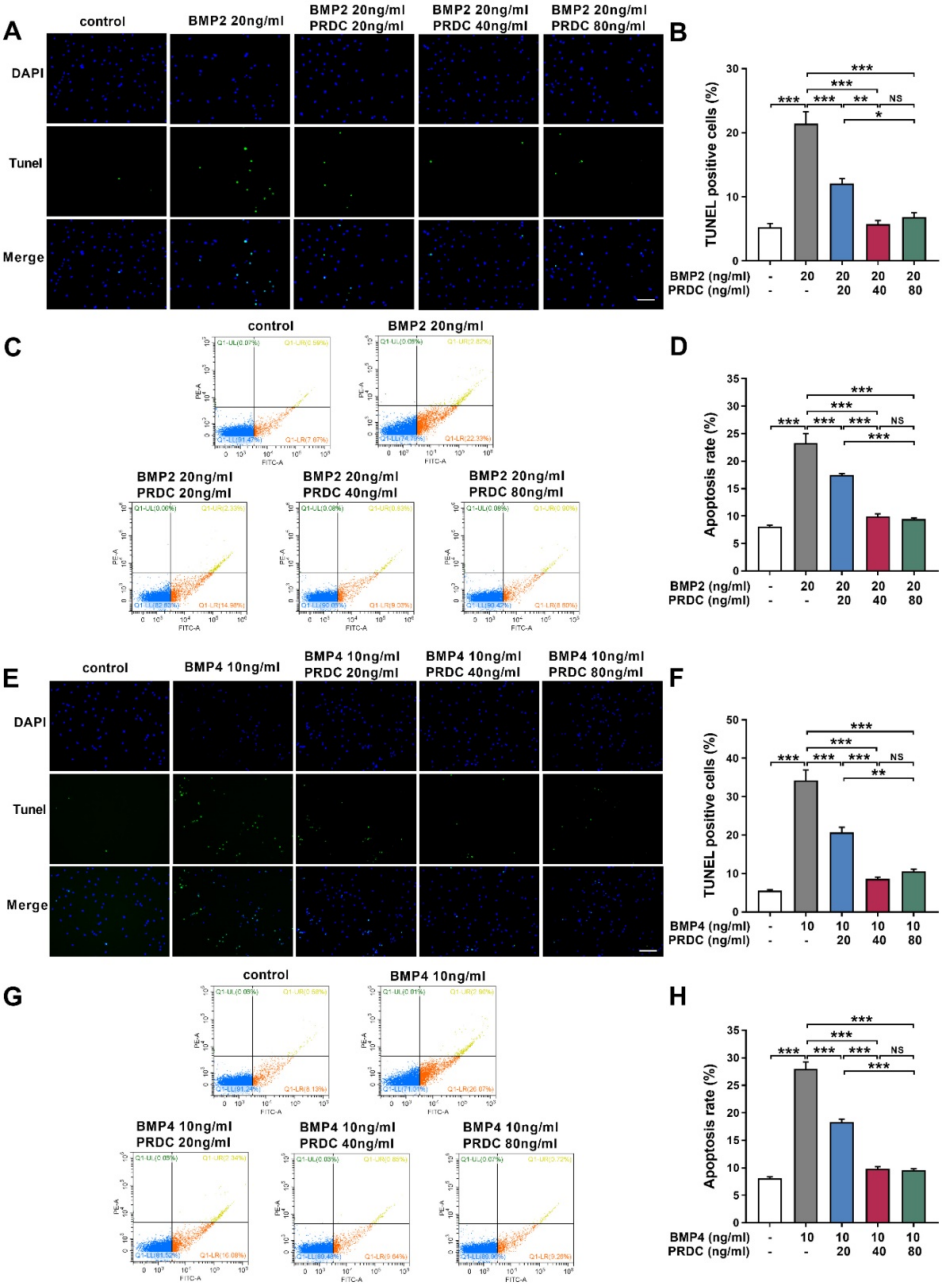
** PRDC antagonized the pro-apoptotic effects of BMP2/4 on dPASMCs. (A-B)** Representative images of the TUNEL assay and quantitative analysis of TUNEL positive in dPASMCs exposed to BMP2 or BMP2+PRDC, Scale bar, 50 µm (n=4). **(C)** Early and late apoptosis of dPASMCs exposed to BMP2 or BMP2+PRDC measured by flow cytometry with Annexin V-FITC/propidium iodide (PI) staining. **(D)** Percentage of apoptosis rate in dPASMCs exposed to BMP2 or BMP2+PRDC (n=4). **(E-F)** Representative images of the TUNEL assay and quantitative analysis of TUNEL positive in dPASMCs exposed to BMP4 or BMP4+PRDC, Scale bar, 50 µm (n=4). **(G)** Early and late apoptosis of dPASMCs exposed to BMP4 or BMP4+PRDC measured by flow cytometry with Annexin V-FITC/propidium iodide (PI) staining. **(H)** Percentage of apoptosis rate in dPASMCs exposed to BMP4 or BMP4+PRDC (n=4). *P < 0.05, **P < 0.01, ***P < 0.001,*^NS^ p* >0.05.

Balance between cell proliferation and apoptosis is essential to the maintenance of homeostasis in dPASMCs. Under baseline control conditions, the percentage of TUNEL labeling-positive dPASMCs was low. BMP2 (20 ng/ml) or BMP4 (10 ng/ml) treatment for 24 h significantly increased the percentage of TUNEL labeling-positive dPASMCs, but PRDC pretreatment significantly decreased the percentage of TUNEL labeling-positive dPASMCs (Figure 7A-B, E-F). Otherwise, flow cytometry analysis of dPASMCs apoptosis indicated that BMP2 (20 ng/ml) or BMP4 (10 ng/ml) treatment led to a dramatically increase in the proportion of dPASMCs apoptosis compared with control condition, but PRDC inhibited the pro-apoptosis effects of BMP2/4 on dPASMCs (Figure 7C-D, G-H).

**Figure 8:**
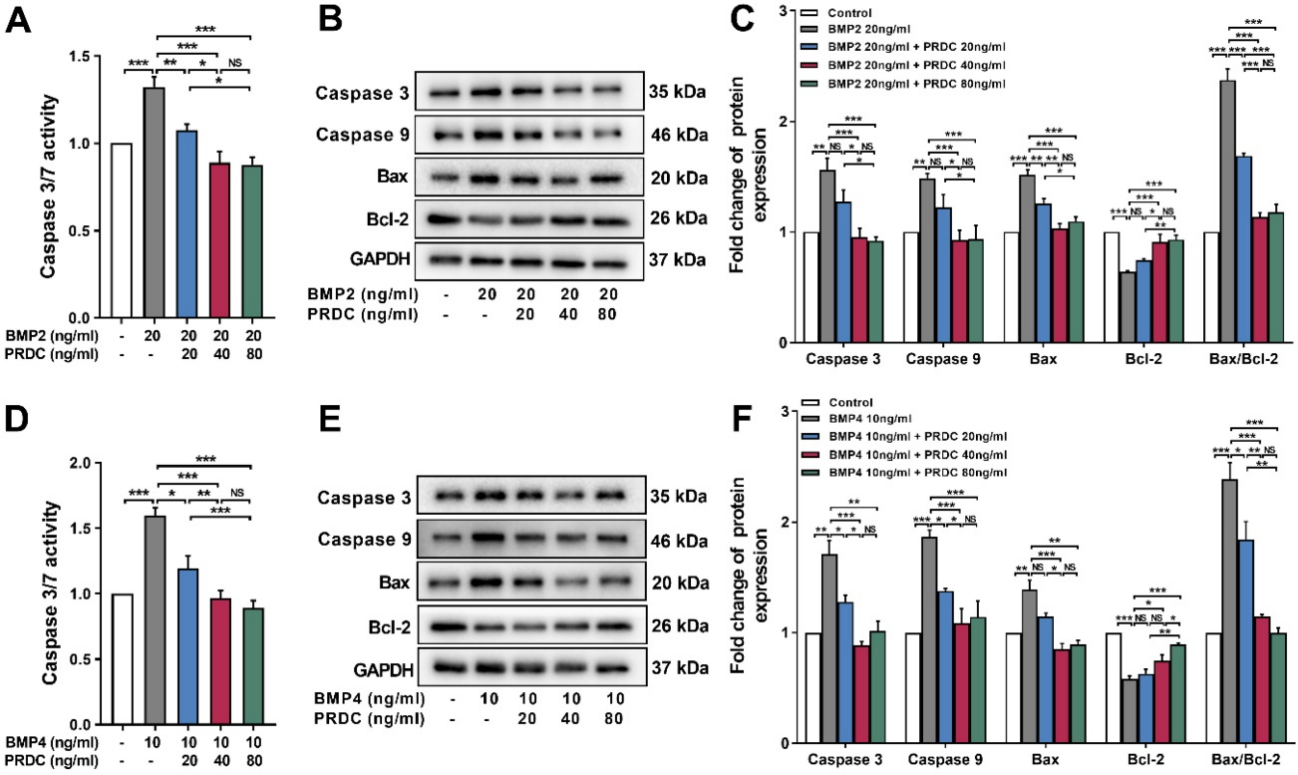
** PRDC restored the expression of apoptosis-associated proteins in dPASMCs induced by BMP2/4. (A)** Caspase3/7 activity in dPASMCs exposed to BMP2 or BMP2+PRDC (n=6). **(B-C)** Representative western-blotting images and densitometry results of caspase3, caspase9, Bax, Bcl-2 and the ratio of Bax to Bcl-2 in dPASMCs exposed to BMP2 or BMP2+PRDC (n=3). **(D)** Caspase3/7 activity in dPASMCs exposed to BMP4 or BMP4+PRDC (n=6). **(E-F)** Representative western-blotting images and densitometry results of caspase3, caspase9, Bax, Bcl-2 and the ratio of Bax to Bcl-2 in dPASMCs exposed to BMP4 or BMP4+PRDC (n=3). *P < 0.05, **P < 0.01, ***P < 0.001,*^NS^ p* > 0.05.

As shown in Figure 8A, D, the activity of caspase3/7 significantly increased in dPASMCs exposed to BMP2 (20 ng/ml) or BMP4 (10 ng/ml) and was restored by PRDC co-treatment to the level observed in control group. Meanwhile, western blot analysis showed that BMP2 (20 ng/ml) or BMP4 (10 ng/ml) treatment up-regulated the expression of apoptosis-associated proteins caspase3, caspase9, Bax, but down-regulated the expression of Bcl-2, thus increased the ratio of Bax to Bcl-2 (Bax/Bcl-2), however, co-treatment with PRDC reversed these changes (Figure 8B-C, E-F).

### Recombinant PRDC supplementation deteriorated the hypertensive status in rats

#### Survival, general status and osmotic pump delivery

No death occurred in control group. Two deaths in MCT-4W group, two deaths in MCT-Saline group and five deaths in MCT-PRDC group were observed. Remaining rats survived to the harvesting time-points and all experienced the right heart catheterization procedure. Derived data of these rats were all utilized in the final statistical analysis. As presented in Table S4, slow weight gain, hydrothorax and ascites were all presented in rats of MCT-Saline group, and these phenomena were further aggravated in rats of MCT-PRDC. Otherwise, osmotic pump delivery failure of PRDC, such as, occlusion of the delivery cannula, residual of PRDC, was not observed at the harvesting time points.

### PRDC supplementation exacerbated pulmonary hemodynamic indices

**Figure 9:**
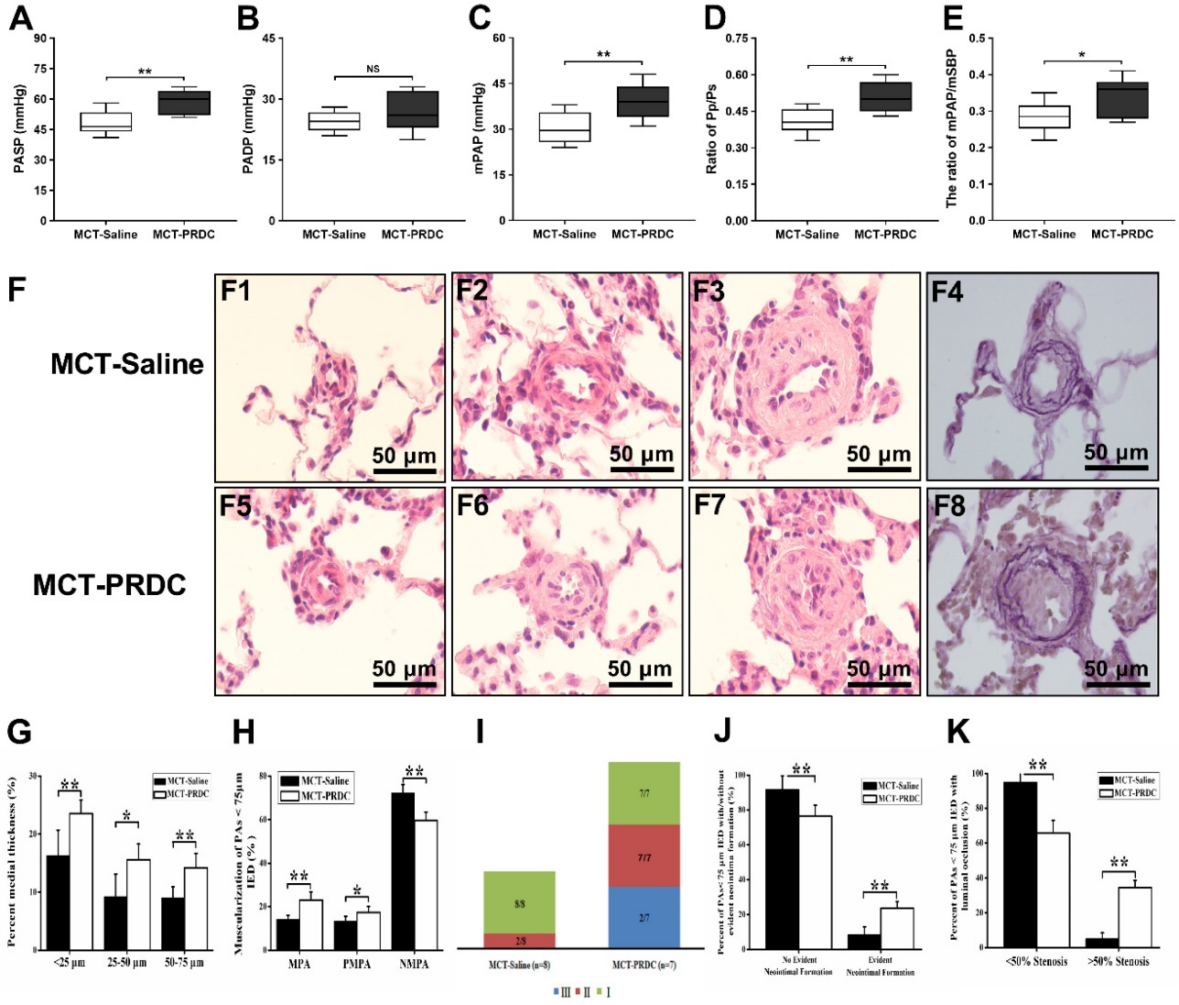
** PRDC administration deteriorated rat pulmonary hemodynamics and pulmonary vasculopathies. (A-E)** Effect of PRDC administration on PASP (A), PADP (B), mPAP (C), Pp/Ps (D), mPAP/mSBP (E). **(F)** Representative morphological imagines from MCT-Saline and MCT-PRDC (hematoxylin-eosin staining, F1-F3, F5-F7, ×1000; Weigert and Van Gieson staining, F4, F8, ×1000); **(G)** Percent medial thickness of PAs < 75 µm IED; **(H)** Distribution of non-, partial- and fully-muscularization of PAs < 75 µm IED; **(I)** Proportion of pulmonary vasculopathies stages in PAs < 75 µm IED; **(J)** Percent of PAs < 75 µm IED with or without neointimal formation in rats with stage II or III pulmonary vasculopathies; **(K)** Percent of PAs <75 µm IED with < 50% or > 50% luminal occlusion in rats with stage II or III pulmonary vasculopathies. MPA, muscularized pulmonary artery; PMPA, partial muscularized pulmonary artery; NMPA, non-muscularized pulmonary artery. *P < 0.05, **P < 0.01, ***P < 0.001,*^NS^ p* > 0.05.

As presented in Figure 9A-E and Figure S2 B-C, the mean values for PASP, mPAP, Pp/Ps and mPAP/mSBP of MCT-Saline all increased to a higher level as compared with those parameters in rats of control group. While, these hemodynamic parameters reached to a much higher level in rats of MCT-PRDC implying PRDC supplementation further deteriorated the hypertensive status induced by monocrotaline injection in rats.

### PRDC supplementation worsened the extent of pulmonary vasculopathies

As presented in Figure 9F, pulmonary vasculopathies of muscularization and cellular intimal proliferation emerged in MCT-Saline, while muscularization, neointimal formation and luminal stenosis presented in MCT-PRDC, concretely, as presented in Table S5 and Figure 9I**,** pulmonary vasculopathies of stage I (8/8), stage II (2/8) and stage III (0/8) by Heath-Edwards classification presented in rats of MCT-Saline, while pulmonary vasculopathies of stage I (7/7), stage II (7/7) and stage Ⅲ (2/7) were observed in rats of MCT-PRDC, indicating that continuous supplementation of PRDC exacerbated the spectra of pulmonary vasculopathies from reversible lesions (muscularization and cellular intimal proliferation) in MCT-Saline to borderline lesions (apparent luminal stenosis) in MCT-PRDC.

### Quantitative analysis of pulmonary vasculopathies of muscularization, cellular intimal proliferation and luminal stenosis

As presented in Figure 9G, lung morphological analysis demonstrated severe medial hypertrophy, but to different extent, in pulmonary arteries (PAs) < 75 μm in external diameter (IED) in MCT-Saline and MCT-PRDC, interestingly, as compared with that of MCT-Saline, medial hypertrophy determined by percent medial wall thickness (MT%) of pulmonary arteries (PAs) < 75 μm in external diameter (IED) reached to a much higher level in rats of MCT-PRDC. Furthermore, a pervasive extension of smooth muscle layer into peripheral pulmonary arterioles < 25 μm IED in MCT-PRDC (Figure 9F5).

Subsequently, the extent of PAs muscularization (<75 μm IED) was also quantitatively evaluated. As presented in Figure 9H, as compared with that of MCT-Saline, the extent of muscularization was all exacerbated in MCT-PRDC as quantitatively analyzed by the percent of PAs < 75 μm IED having full or partial muscular wall. Apparently, the decrease in the proportion of normally non-muscularized PAs and the increase in the proportion of fully/partially-muscularized PAs observed in rats of MCT-Saline were further enhanced in rats of MCT-PRDC, implicating continuous PRDC supplementation incurred much more extensive muscularization in PAs < 75 μm IED than that of MCT-Saline.

Furthermore, as presented in Figure 9J, in rats (2/8) of MCT-Saline with pulmonary vasculopathy of stage Ⅱ by Heath-Edwards classification, the percent of PAs < 75 μm IED with cellular intimal proliferation was 8.26± 4.75%, while in rats (7/7) of MCT-PRDC with pulmonary vasculopathy of stage Ⅱ by Heath-Edwards classification, the percent of PAs < 75 μm IED with evident neointima was 23.58 ±3.87%.

Moreover, as showed in Figure 9K, in rats (2/8) with pulmonary vasculopathy of stage Ⅱ of MCT-Saline group, quantitative analysis found that cellular intimal proliferation in PAs < 75 μm IED only resulted into < 50% luminal occlusion and no > 50% luminal occlusion, while in rats (7/7) with pulmonary vasculopathy of stage II of MCT-PRDC group, PAs < 75 μm IED with evident neointima not only led to < 50% luminal occlusion but also > 50% luminal occlusion, indicating that continuous PRDC supplementation incurred much more apparent luminal stenosis in PAs < 75 μm IED than that of MCT-Saline.

### PRDC administration accentuated right ventricle hypertrophy and function

**Figure 10:**
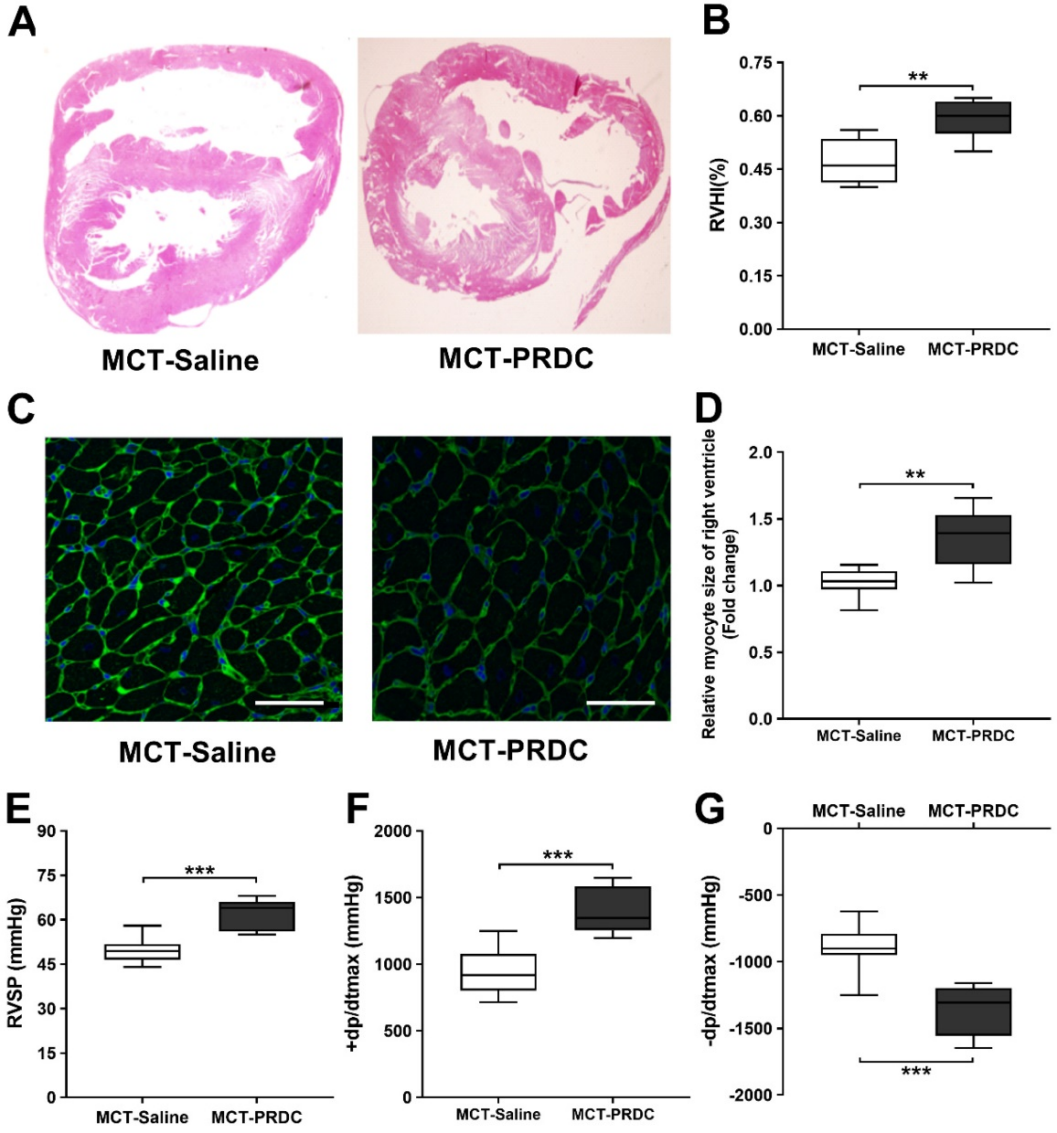
** PRDC administration aggravated the right ventricular hypertrophy and function induced by monocrotaline injection in rats. (A)** H&E staining of rat heart transversal section; **(B)** Quantitative analysis of right ventricular hypertrophy index (RVHI); **(C)** WGA staining of rat heart (bar=50 µm); **(D)** Quantitative analysis of right ventricular cardiac myocytes by WGA staining; **(E)** Effect of PRDC administration on RVSP; **(F-G)** Quantitative analysis of right ventricular contraction function (+dp/dtmax, F) and relaxation function (-dp/dtmax, G). *P < 0.05, **P < 0.01, ***P < 0.001,*^NS^ p* > 0.05.

As presented in Figure 10, at the 4^th^ week after monocrotaline injection, as compared with rats in MCT-Saline, the extent of right ventricular hypertrophy (Figure 10A-D) and cardiac function (±dp/dtmax, Figure 10E-G) were further accentuated in rats of MCT-PRDC, indicating PRDC administration significantly aggravated the secondary compensatory responses of right ventricle in terms of RV hypertrophy and deteriorated RV function.

## Discussion

The accurate mechanism for PAH is still unclear, effective therapies are still absent to reverse pulmonary vascular remodeling and improve the long-term survival of patients with PAH. Previous studies have confirmed that, as the major constituent of the media layer of pulmonary vasculature, dPASMCs regaining of the proliferative, migratory and apoptosis-resistant potential contributed to the hypertrophy of pulmonary media, muscularization of pulmonary arteries and narrowing of pulmonary arterial lumen 1, 8. Therefore, finding targets, which could regulate these abnormal biological behaviors of dPASMCs observed in PAH, would be our subsequent research strategy. This study for the first time confirmed the down-regulation of PRDC in hypertensive lungs, investigated the role of PRDC in dPASMCs phenotype switch and monocrotaline-induced PAH in rats.

### PRDC and BMP signaling were both downregulated in hypertensive lungs

PRDC is a secreted glycoprotein, which belongs to the BMP antagonist family 9. BMPs are a group of factors that belong to the TGF-β superfamily, which play critical roles in regulating cell proliferation, apoptosis, differentiation and tissue development 17-18. BMPs usually interact with membrane receptors and trigger Smads phosphorylation to translate the signals into the nucleus and regulate target gene expression (Id) 19-20. Interestingly, misregulation of BMP ligands, receptors and the downstream signaling has been reported to be deeply involved into the genesis of PAH 21-22, so, theoretically, PRDC may exert its role via BMP cascade. Research in reproductive system showed that PRDC specially blocked BMP-2 and BMP-4 signal cascade *in vitro*, but not that of TGF, GDF9 or Activin 10-11, so, it was proposed that PRDC expression in granulosa cells in mouse ovaries might antagonize BMP signaling regulating follicle development and luteinization 11. Recent studies further confirmed that PRDC was expressed in lung tissues during embryonic development 12, however, little is known about the role of PRDC in embryogenesis or under physiological and pathological conditions in the adult, let alone in the condition of PAH.

This study found that PRDC level was significantly decreased in hypertensive lungs of patients with IPAH. However, in terms of the original data as shown in Table S1-S2, the extent of PAH of these 6 people with IPAH is all serious and the degree stratification of PAH is not apparent. Therefore, if only analyzing the correlation between lung PRDC band density and the hemodynamic indices of patients with IPAH obtained from right heart catheterization (RVSP, PASP, mPAP, CO, CI, PVR and TPVR), no statistical difference were found (Figure S4), however, without the data of control subjects, these results may not truly reflect their relationship. Interestingly, when the values within the normal range were assumed to the 6 subjects in the control group (PASP 25 mmHg, mPAP 15 mmHg, RVSP 28 mmHg, CO 5 L/min, CI 3 L/min·m^2^, PVR 2.5 Wood Units, TPVR 3 Wood Units), reanalyzing results demonstrated apparent negative correlation between lung PRDC band intensity and the degree of PAH (RVSP, PASP, mPAP, CO, CI, PVR and TPVR) (Figure S5), however, the concrete values for RVSP, PASP, mPAP, CO, CI, PVR and TPVR of the 6 subjects in the control group are not available, so, in consideration of the lack of pulmonary hemodynamic indices of subjects in control group, these analysis results of Figure S5 only inferred some speculating implication.

Fortunately, right heart catheterization was performed on all the rats surviving to the harvesting time-points, so the hemodynamic indices including RVSP, PASP, mPAP, and the extent of right ventricular hypertrophy (RVHI) were all obtained. So the relationship between lung PRDC band density and the severity of PAH (RVSP, PASP, mPAP and RVHI) was successfully and entirely demonstrated in rats. As demonstrated in Figure 2D, in MCT-PAH rats, linear regression analysis demonstrated that lung PRDC band intensity negatively correlated with the severity of MCT-PAH as measured by RVSP, PASP, mPAP, and RVHI, which naturally presented a necessary supplement for the deficiency of human data presented in Figure 1.

Consistently, immunofluorescent staining further demonstrated robustly expressed in the endothelium and medium of normal PAs from normal human lungs and rats of control group, but weakly expressed in remodeled PAs of hypertensive lungs (Figure 1D, E and Figure 2E, F), thus, this study for the first time described the distribution and location of PRDC in normal lungs and its change in hypertensive lungs.

### PRDC was deeply involved into the phenotype transformation of dPASMCs

#### PRDC promotes dPASMCs proliferation

Differentiated dPASMCs reenter cell cycle is a key event of PAR, which referred to cell cycle transition of dPASMCs from G0/G1 phase to S phase and G2/M phase 1. The cell cycle is a highly complex, ordered system of biochemical transitions, which is precisely regulated by a series of cyclin dependent kinase (CDK) complexes and CDK-inhibitory proteins 23. In accordance with previous studies 7, this study demonstrated the proliferation-suppressive effect of BMP2/4 on dPASMCs, but no research reported the effects of PRDC on the proliferation-suppressive effects of BMP2/4 on dPASMCs, let alone the downstream signaling.

As demonstrated in Figure 3, BMP2 (20 ng/ml), BMP4 (10 ng/ml) had the optimal inhibitory effects on the proliferation of dPASMCs, but a further increase in BMP2/4 concentration did not enhance the inhibitory effect on dPASMCs proliferation. Firstly, this phenomenon indicated that the proliferation inhibitory effect of BMP2/4 on dPASMCs doesn't always present a linear relationship with dose, and this is in accord with previous researches 7, 24; Secondly, although BMP2/4 of higher dose still showed an inhibitory effect on dPASMCs proliferation, this inhibitory effect was not as effective as that of BMP2 (20 ng/ml), BMP4 (10 ng/ml). Previous researchers found that BMP2 could decrease TRPC expression, store-operated Ca^2+^ entry, and basal [Ca^2+^]_i_ and inhibit the proliferation and migration of rat dPASMCs, while BMP2 could up-regulate the expression and function of voltage-gated K^+^ channels in dPASMCs, which subsequently exerted proapoptotic and/or antiproliferative effects on dPASMCs 25-26, furthermore, BMP4 induces HO-1 via a Smad-independent, p38MAPK-dependent pathway in dPASMCs 27-28, which all implicated that BMP2/4 of higher dose may also exert their biological effects in a BMP cascade independent way.

Interestingly, studies in osteogenesis found that PRDC blocked the BMP2/4 effect via BMP-Smad1/5/8 pathway 7, 29. Present study firstly confirmed that PRDC antagonized the anti-proliferative effects of BMP2/4 on dPASMCs (Figure 3). Furthermore, as demonstrated in Figure 4, pretreatment with PRDC negatively regulated the expression of CDK-inhibitor p21 and p27, but had no significant effect on CDK cyclin D1 in dPASMCs, suggesting that PRDC promoted dPASMCs proliferation partially through the regulation of p21 and p27. Those findings revealed that BMP2/4 treatment was able to inhibit dPASMCs proliferation, while PRDC treatment could promote the proliferation in dPASMCs by antagonizing BMP 2/4.

### PRDC promotes dPASMCs migration

dPASMCs migration plays vital roles in the muscularization, neointima formation of distal pulmonary arteries/arterioles 1. Actually, dPASMCs migration is an organized sequence of events. In normal pulmonary arteries, extracellular matrix (ECM) locally produced by pulmonary vascular cells maintain the non-migratory status of dPASMCs, while, dPASMCs migration is controlled by the balance of matrix metalloproteinases (MMPs) and tissue inhibitors of metalloproteinases (TIMPs) in ECM, which is confirmed to be responsible for the turnover of ECM components and the fragmentation of the internal elastic lamina, a prominent feature of PAR in PAH 1. Actually, MMPs in ECM exist in inactive type and their activity is counterbalanced by TIMPs 30-31. Increased MMPs activity modulated SMC migration and incurred neointimal formation after vessel injuries, while, transfer of TIMP-1 gene inhibited SMC migration and neointimal formation in saphenous vein 31-32.

Previous researches confirmed that perturbation of the balance between MMPs and TIMPs in hypertensive lungs is closely related to dPASMCs migration and contributed to the progression of PAH 1, 30, 33-35. Present study observed that BMP2/4 suppressed dPASMCs migration accompanied by a down-regulation of the expression of MMP2 and MMP9, up-regulation in the expression of TIMP1 and TIMP2, and for the first time, we also found that PRDC promoted dPASMCs migration by antagonizing the migration suppressive effect of BMP2/4 on dPASMCs via up-regulating MMP2/9 and down-regulating TIMP1/2 expression, suggesting that PRDC enhanced the migration of dPASMCs partially through disrupting the balance of MMPs and TIMPs expression (Figure 5,6).

### PRDC inhibits dPASMCs apoptosis

Apoptosis is an important cytological event which maintains the functional and structural integrity of pulmonary vasculature 36-37, while, disturbing the balance between dPASMCs proliferation and apoptosis incurred the emergence of apoptosis-resistant dPASMCs that exert indispensable roles in PAR 1, 36. Bcl-2 family proteins mediated mitochondrial apoptotic pathway has central regulatory roles in the early phase of cell apoptosis 1, 38. Bcl-2 mRNA has been shown to be up-regulated in lung tissues from patients with IPAH and family PAH 39-40. And overexpression of Bcl-2 inhibits staurosporine-induced apoptosis in dPASMCs 41. Previous studies reported that BMP2/4 exerted vital roles in the lung morphogenesis, development and function via affecting the dynamic balance between dPASMCs proliferation and death, furthermore, the proapoptotic effects of BMP2/4 on dPASMCs could be mediated by activation of caspases-3, -8, and -9, cytochrome c release, and down-regulation of Bcl-2 7. Present study demonstrated that BMP2/4 increased the ratio of Bax to Bcl-2, activated caspase 3/9 and triggered the process of cell apoptosis (Figure 7, 8), indicating their pro-apoptosis effects on dPASMCs, while PRDC could significantly reverse the pro-apoptosis effect of BMP2/4 on dPASMCs. This study for the first time confirmed that PRDC could inhibit dPASMCs apoptosis triggered by BMP2/4, at least, partially through modulating Bcl-2 and caspase family proteins.

### PRDC alone has no direct roles on the proliferation, migration and apoptosis of dPASMCs

In the initial design of this study, the direct effect of PRDC with gradient dose (0 ng/ml, 10 ng/ml, 20 ng/ml, 40 ng/ml, 80 ng/ml, 160 ng/ml, 320 ng/ml) on the phenotype change of dPASMCs (proliferation, migration and apoptosis) were investigated. However, as demonstrated in Figure 3C, no direct effects of PRDC alone were observed on dPASMCs proliferation. Furthermore, as presented in Figure S6A, B, PRDC alone, even at the concentration of 320 ng/ml, still has no direct effect on the migration and apoptosis of dPASMCs.

Previous researches on the biological activity of PRDC and our results in this studies all confirmed that PRDC exerts its roles via antagonizing the BMP cascade 9, 11, 14, and no studies have investigated the direct effect of PRDC on dPASMCs, let alone the phenotype transform of dPASMCs under the condition of PAH. This is the first study focused on the direct role of PRDC on dPASMCs phenotype and results in this study concluded that no apparent effects of PRDC alone were observed on dPASMCs proliferation, migration and apoptosis, however, interpretation and extrapolation of the reference value of these results should be cautious and injudicious in terms of different types of cell and different pathophysiological states.

### PRDC administration exacerbated monocrotaline-induced hypertensive status in rats

No research has investigated the role of PRDC in PAH of any types, so detailed evaluation of the *in vivo* experiment was made in multiple aspects. As compared with that of rats in MCT-Saline, rats in MCT-PRDC presented higher rate in death, hydrothorax, ascites, and lower weight gain, indicating PRDC administration deteriorated the survival and general status in rats with hypertensive status.

As for hemodynamic indices of pulmonary circulation, the mean values for RVSP, PASP, mPAP observed in rats of MCT-PRDC further increased to a higher level comparing with those of MCT-Saline (Figure 9A-E). As to pulmonary vasculopathies, apparently more partial-muscularized PAs and non-muscularized PAs advanced into fully muscularized PAs, and remuscularization of pulmonary arterioles also emerged in rats of MCT-PRDC (Figure 9F-H). Furthermore, more remodeled PAs with neointimal formation leading to near luminal stenosis presented in MCT-PRDC, which were seldomly found in MCT-Saline (Figure 9J, K). With regard to right ventricle, the extent of RV hypertrophy (Figure 10A-D), systolic function (+dp/dtmax, Figure 10F) and diastolic function (-dp/dtmax, Figure 10G) further deteriorated in rats of MCT-PRDC comparing with those of MCT-Saline. These results elicited the conclusion that the hypertensive status observed in MCT-Saline was further progressed by additional PRDC administration into a much more severe condition in terms of death rate, general status, pulmonary hemodynamics, pulmonary vasculopathies, RV hypertrophy and function.

However, the true potential of PRDC administration on monocrotaline-induced PAH is still not completely ascertained. First of all, the dose of 15 μg is based on our previous pre-exploration experiments (Table S6). Because the fertility and reproduction capacity of the PRDC gene rats constructed in the previous stage were severely restricted 42, so the approach of supplementing the PRDC recombinant protein was adapted to backward infer the influence of lung PRDC downregulation on PAH: delay, reversal or no effect? Hitherto no studies have investigated the role of PRDC in PAH. Only parallel data from our previous reports on DAN (another BMP antagonists) and structure insights of PRDC gave us some limited referential value in the design of *in vivo* experiment protocol 7, 9, so preliminary experiments on PRDC administration with gradient doses were explored. Concretely, rats of MCT-4W were divided into 5 groups of PRDC 0 μg (n=5), 1.875 μg (n=5), 3.75 μg (n=5), 7.5 μg (n=5), 15 μg (n=5). Results from preliminary experiments indicated that PRDC administration aggravated the extent of PAH phenotype of MCT-4W+PRDC 15 μg to a similar level of MCT-5W in terms of general condition, pulmonary vascular remodeling, pulmonary hemodynamic indices and right ventricular hypertrophy, indicating signs of concentration-dependent enhancement of PRDC administration on PAH.

Based on these early findings, dose of PRDC 15 μg was chosen as the follow-up *in vivo* experimental dose. Furthermore, normal rats (n=5) was also received PRDC of 15 μg and right heart catheterization were also performed 4 weeks later (data not shown). Subsequent data analysis confirmed that supplementation dose of 15 μg PRDC administration had no obvious effects on the general status, pulmonary hemodynamics and right ventricular hypertrophy of normal rats, which means that under normal circumstances, only supplementation of PRDC cannot affect pulmonary hemodynamics, and it also excludes the toxic effect of PRDC of 15 μg on normal rats.

Some limitations of the present study should be clarified. This *in vivo* study is performed on monocrotaline-induced PAH in rats and this inflammatory type of PAH presented major difference in the initial and progression with hypoxia PAH and systemic-to-pulmonary shunt induced PAH 43, so it should be prudent and cautious to interpret and extrapolate the results presented in this study.

**Figure 11:**
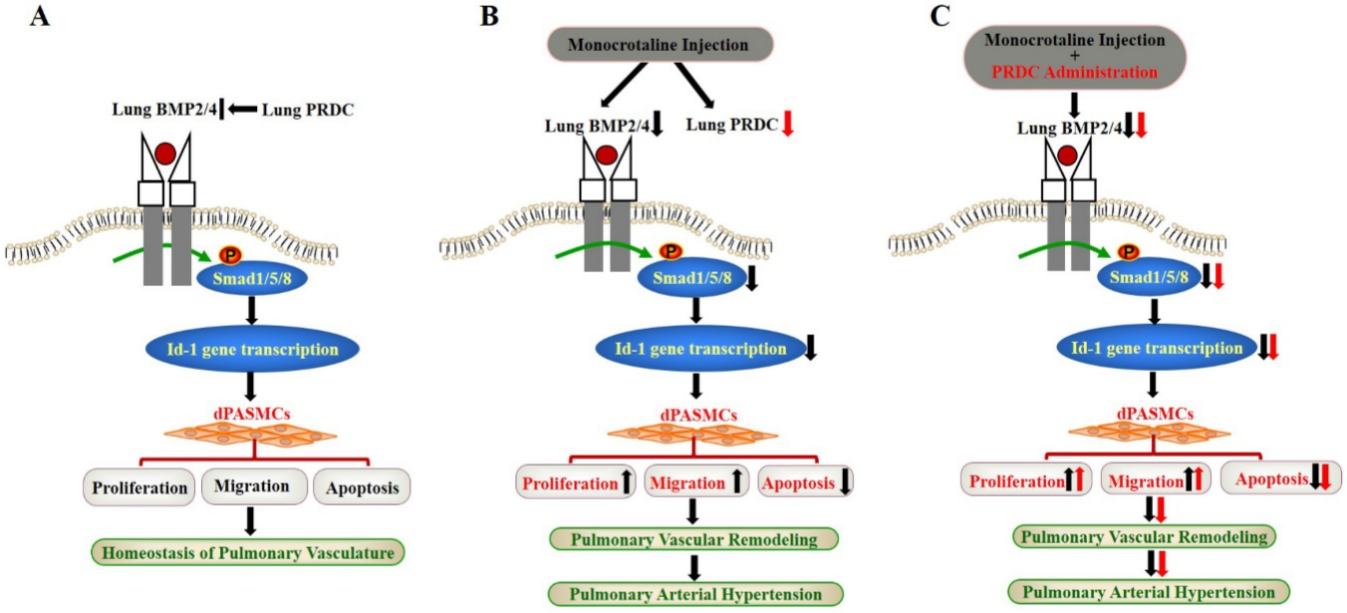
** Schematic diagram of findings of this study. (A)** Lung PRDC physiologically antagonizes lung BMP cascade to maintain the homeostasis of pulmonary vascularture; **(B)** Monocrotaline-induced suppression of lung BMP cascade resulted into the phenotype transformation of dPASMCs, pulmonary vascular remodeling and PAH; **(C)** PRDC administration further exacerbated monocrotaline-induced PAH.

Taken together, for the first time, our findings demonstrated that PRDC promoted the phenotype transformation of dPASMCs via antagonizing BMP cascade, while, as verified by the disproofs derived from the *in vivo* PRDC administration experiment in rats, PRDC down-regulation in hypertensive lungs retarded the progression of PAH (Figure 11).

## Supplementary Material

Supplementary methods, figures and tables.

## Abbreviations

PAH: pulmonary arterial hypertension
IPAH: idiopathic pulmonary arterial hypertension
BMP: bone morphogenetic protein
BMPR2: BMP receptor 2
MCT: monocrotaline
RVSP: right ventricular systolic pressure
PASP: pulmonary arterial systolic pressure
mPAP: mean pulmonary arterial pressure
RVHI: right ventricular hypertrophy index
MPA: muscularized pulmonary artery
PAR: pulmonary arterial remodeling
PMPA: partial muscularized pulmonary artery
NMPA: non-muscularized pulmonary artery
RHC: Right heart catheterization
dPAs: distal pulmonary arteries
MT%: the percentage of medial wall thickness
dPASMCs: distal pulmonary artery smooth muscle cells
PCNA: proliferating cell nuclear antigen
CDK: cyclin dependent kinase
MMPs: matrix metalloproteinases
TIMPs: tissue inhibitors of metalloproteinases
SMCM: smooth muscle cell medium
