# Supplementary material for: Compensatory roles of Protein Related to DAN and Cerberus (PRDC) decrease in pulmonary arterial hypertension
Source: Int J Biol Sci. 2022 Mar 6;18(6):2372–91. doi: 10.7150/ijbs.70247 (PMC8990474; doi:10.7150/ijbs.70247)
Supplement: Supplementary file 1 — Supplementary methods, figures and tables. [file ijbsv18p2372s1.pdf]

Supplementary methods

1. Cell cycle distribution detection

dPASMCs were cultured in six-well plates and subjected to resting SMCM (containing 0.2% FBS) for 24 h to synchronize growth. Next, cells were stimulated with complete SMCM (containing 2% FBS) for another 12 h and 24 h. After that, cells (1×10^6 cells/ml) were harvested and fixed gently with 75% ethanol at -20°C overnight. Then cells were washed with phosphate-buffered saline (PBS) twice, centrifuged at 800 rpm for 3 min and resuspended in a staining solution buffer. After treatment with RNaseA (100 U/ml) for 30 min, dPASMCs were stained with PI (2 mg/ml) and then analyzed using a flow cytometry (BD Biosciences, New Jersey, USA). The proportion of cells in the G0/G1, S and G2/M phases was determined using ModFitLT software.

2. Elisa assay

Rat PRDC Elisa kit was purchased from Sigma-Aldrich (Mouse PRDC Elisa kit, Cat no: RAB0838). Briefly, 100 µl of each standards and cell supernatant samples were added into appropriate wells and incubated for 2.5 h at room temperature. After washing with wash solution twice, 100 µl of detection antibody was added to each well and incubated for 60 min at room temperature with gentle shaking. After washing with wash solution twice, 100 µl of streptavidin solution was added and incubated for 45 min at room temperature with gentle shaking. And then, washing with wash solution twice, 100 µl of TMB one-step substrate reagent was added and incubated for 30 min at room temperature in the dark, finally, 50 µl of stop solution was added to terminate the reaction. Absorbance was immediately read at 450 nm, then, standard curve was drawn and PRDC concentration in the cell supernatant was calculated.

Supplementary figures

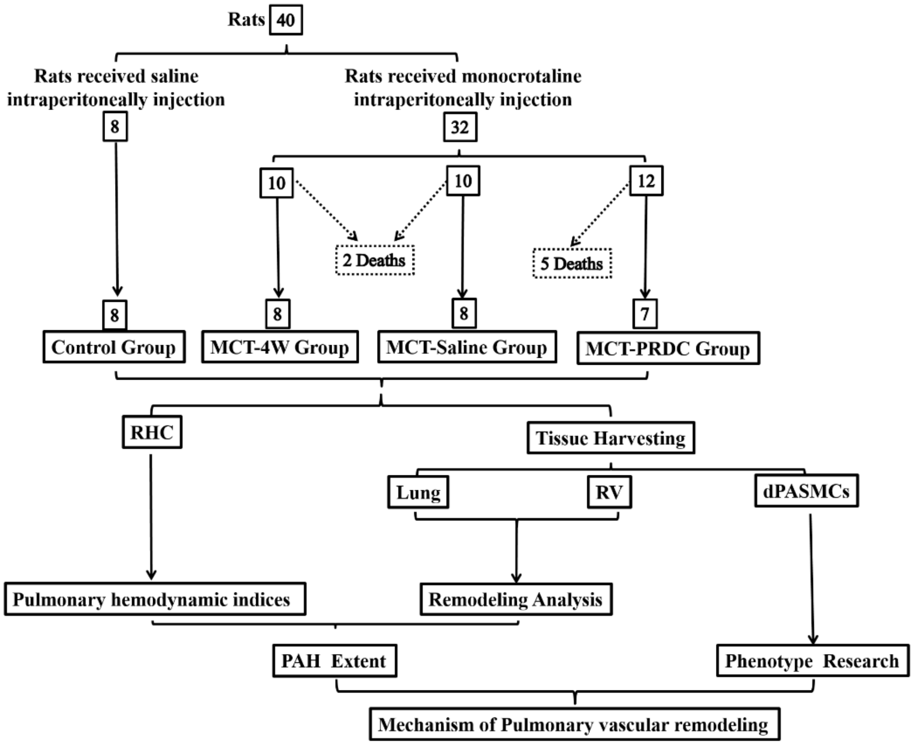

Figure S1. Flow chart of the whole protocol of this study. RV, right ventricle; RHC, right heart catheterization.

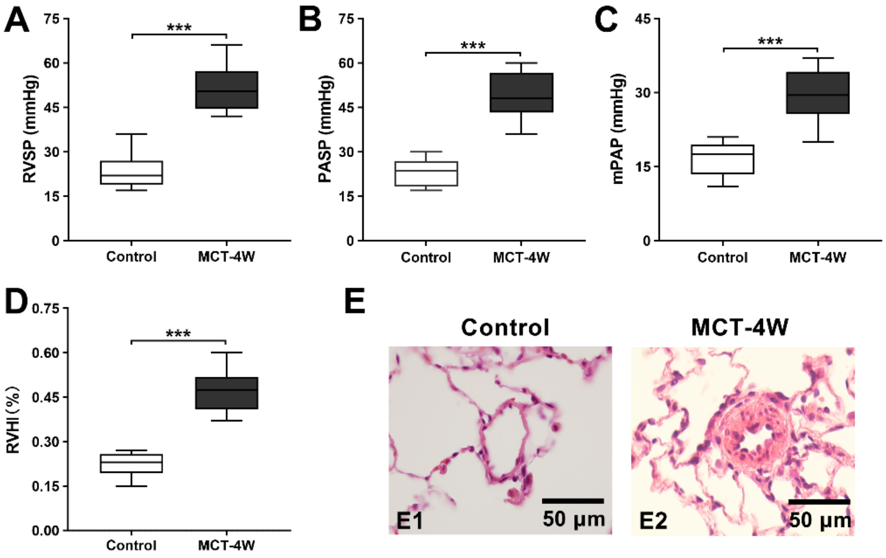

Figure S2. Monocrotaline injection for 4 weeks successfully induced a hypertensive status in rat lungs. A-D, RVSP (A), PASP (B), mPAP (C) and RVHI (D) increased after monocrotaline injection (n=8). E, Normal pulmonary artery from control group (E1); muscularized pulmonary artery from MCT-4W (E2). *$*P < 0.05$, $**P < 0.01$, $***P < 0.001$, $^{NS}p > 0.05$.*

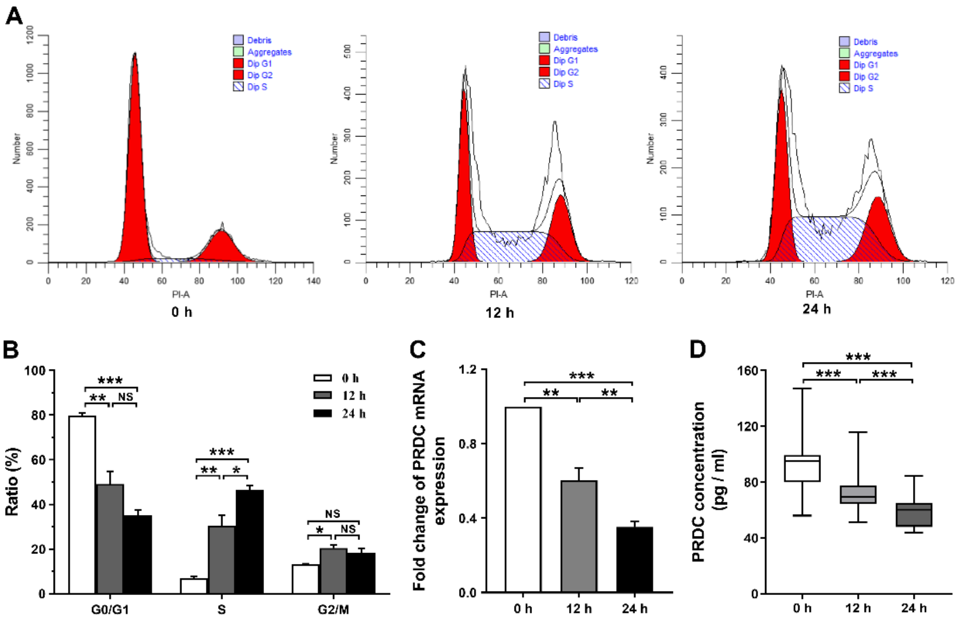

Figure S3. Expression and secretion of PRDC in dPASMCs. A, Distribution of dPASMCs in cell cycle phase detected by flow cytometry. B, Quantitative analysis of dPASMCs distribution in cell cycle phase at indicated time points (n=3). C, mRNA level of PRDC in dPASMCs at indicated time points (n=5). D, PRDC concentration in the supernatants at indicated time points. **P* < 0.05*, ***P* < 0.01,* ****P* < 0.001, ^{NS} *p* > 0.05.*

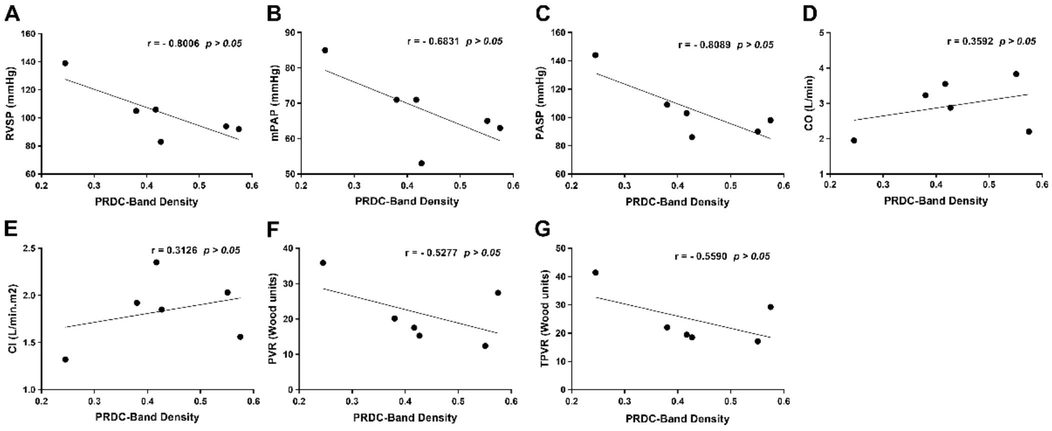

Figure S4. The correlation of PRDC band density in hypertensive lungs with RVSP, PASP, mPAP, CO, CI, PVR and TPVR in patients with IPAH.

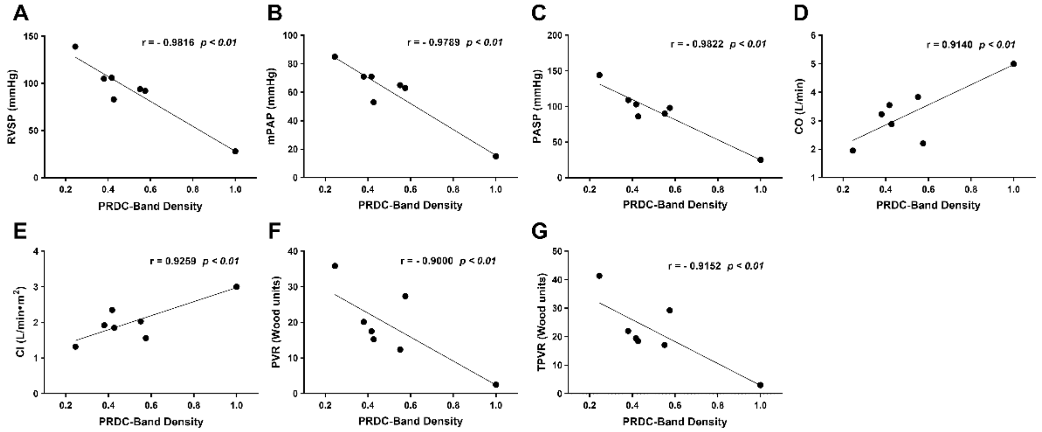

Figure S5. The correlation of lung PRDC band density with RVSP, PASP, mPAP, CO, CI, PVR and TPVR of control subjects and patients with IPAH.

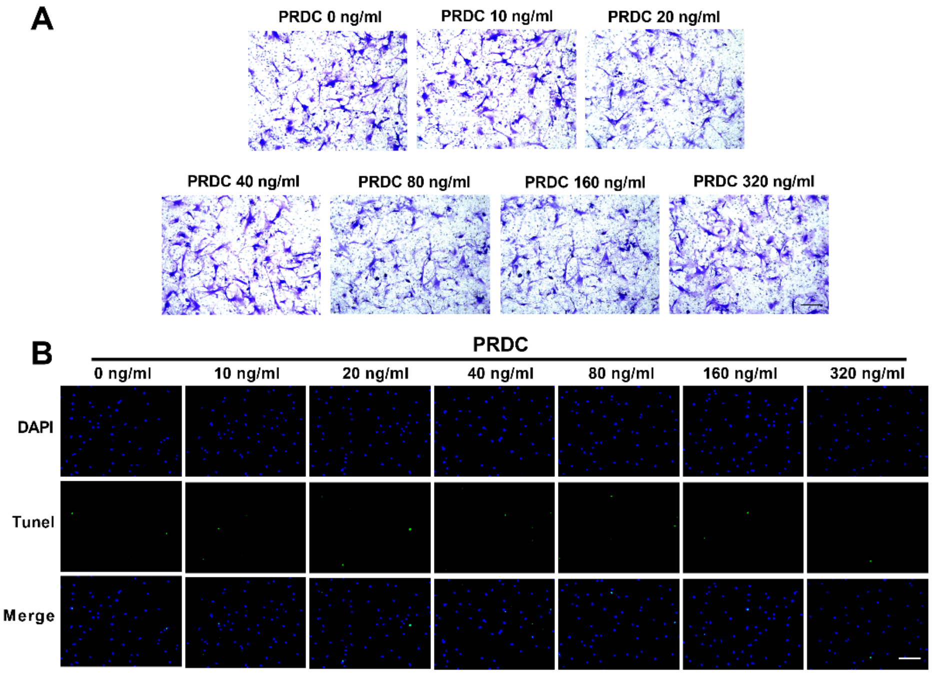

Figure S6. Direct effects of PRDC alone on dPASMCs migration and apoptosis. (A) Representative images of dPASMCs migrated and attached to the membrane bottom surface of transwell chamber exposed to gradient dose of PRDC. (B) Representative images of the TUNEL assay in dPASMCs exposed to gradient dose of PRDC, Scale bar, 50 µm.

Supplementary tables

Table S1. Characteristics of the patients with IPAH and control subjects involved into the preliminary study

**Grouping**	Age (Y)	Gender (F/M)	Aetiology of Surgery	NYHA	BMPRII Mutations Status	Medications at Transplantation
**Control**						
**1**	48	F	Lung cancer	I	N	
**2**	59	M	Lung cancer	I	N	
**3**	62	F	Lung cancer	I	N	
**4**	56	M	Lung cancer	I	N	
**5**	43	F	Lung cancer	I	N	
**6**	35	M	Lung cancer	I	N	
**PAH Patients**						
**1**	48	F	IPAH	IV	N	Sidenafil/ Bosentan
**2**	19	M	IPAH	IV	N	Tadalafil/ Bosentan
**3**	41	F	IPAH	IV	N	Tadalafil/ Bosentan
**4**	16	M	IPAH	IV	N	Tadalafil /Macitentan
**5**	33	M	IPAH	IV	N	Sidenafil /Macitentan
**6**	26	F	IPAH	IV	N	Sidenafil/ Bosentan

M, Male; F, Female; IPAH, idiopathic PAH

Table S2. Characteristics of the patients with IPAH involved into the preliminary study

**Grouping**	Pa. No.1	Pa. No.2	Pa. No.3	Pa. No.4	Pa. No.5	Pa. No.6
**Hemodynamic Indices**						
RVSP (mmHg)	92	106	139	83	94	105
PASP (mmHg)	98	103	144	86	90	109
mPAP (mmHg)	63	71	85	53	65	71
CO (L/min)	2.2	3.55	1.95	2.88	3.83	3.23
CI (L/min/m^2)	1.56	2.35	1.32	1.85	2.03	1.92
PVR (Wood)	27.36	17.51	35.87	15.27	12.37	20.13
TPVR (Wood)	29.23	19.44	41.36	18.47	17.11	21.99
**PRDC Band Density**	0.575	0.417	0.245	0.427	0.551	0.380

IPAH, idiopathic PAH; RVSP, right ventricular systolic pressure; PASP, pulmonary arterial systolic pressure; mPAP, mean pulmonary arterial pressure; CO, cardiac output; CI, cardiac index; PVR, pulmonary vascular resistance; TPVR, total pulmonary vascular resistance

Table S3. Primer Sequences for Real-Time PCR

Gene	Forward 5’-3’	Reverse 5’-3’
PRDC (h)	TTTCCCTGTCCTTGTTCCTG	TGCACCAGTCACTCTTGAGG
GAPDH (h)	GAGTCAACGGATTTGGTCGT	GACAAGCTTCCCGTTCTCAG
PRDC (r)	GGAGGACTCCTTCCAATCCTG	TGTCGGAGTCACTCAGGTTCA
BMPR2 (r)	CCGGGCAGGATAAATCAGGA	ATTCTGGGAAGCAGCCGTAG
Id-1(r)	GAACCGCAAAGTGAGCAAGG	GAACCGCAAAGTGAGCAAGG
GAPDH (r)	GGCACAGTCAAGGCTGAGAATG	ATGGTGGTGAAGACGCCAGTA

(h) Homo sapiens, (r) Rattus norvegicus

Table S4. Survival and general status of rats in this study

**Group**	**Weight (g)**	**Death**	**hydrothorax**	**ascites**	**Survival**
**Control group**					
(n=8)	443.47±22.57	0	0/8	0/8	8
**MCT-4W group**					
(n=10)	367.43±34.59	2	2/8	3/8	8
**MCT-Saline group**					
(n=10)	356.47±39.52	2	3/8	4/8	8
**MCT-PRDC group**					
(n=12)	294. 34±21.93	5	5/7	6/7	7

All data are showed as Mean ± SD. MCT-4W group, monocrotaline injection and raised for 4 weeks group; MCT-Saline, monocrotaline injection and saline administration group; MCT-PRDC, monocrotaline injection and PRDC administration group.

Table S5. Stage of pulmonary vasculopathies by Heath-Edwards classification in pulmonary arteries < 75 μm in external diameter

**Stage of Pulmonary Vasculopathies by Heath-Edwards Classification**	**MCT-Saline group (n=8)**	**MCT-PRDC group (n=7)**
Stage I	8/8	7/7
Stage II	2/8	7/7
Stage III	0/8	2/7

Stage I: Medial hypertrophy;

Stage II: Medial hypertrophy with intimal proliferation;

Stage III: Medial hypertrophy with neointimal formation, luminal stenosis or luminal occlusion.

Table S6. Survival and general status of rats in the preliminary study

**Group**	**Weight (g)**	**Death**	**Hydrothorax**	**Ascites**	**Survival**	**RVSP**	**PASP**	**mPAP**	**RVHI**
**Control group**									
Control-Saline (n=5)	483.56±25.73	0	0	0	5	26.38±4.73	25.08±5.32	17.57±2.35	22.63±5.38
Control-PRDC (n=5)	460. 35±19.47	0	0	0	5	23.86±6.82	22.79±6.17	16.18±3.68	25.03±4.51
**MCT-4W**									
PRDC 0 μg (n=5)	335.64±20.65	1/5	1/5	1/5	4/5	48.62±7.64	46.37±5.36	28.83±5.05	46.25±5.98
PRDC 1.85 μg (n=5)	352.82±35.46	1/5	1/5	2/5	4/5	45.34±5.36	44.15±6.56	26.93±3.24	43.69±3.24
PRDC 3.75 μg (n=5)	326.18±16.89	2/5	2/5	2/5	3/5	50.27±8.43	49.35±6.74	31.88±2.82	49.53±3.36
PRDC 7.5 μg (n=5)	286. 76±18.37	2/5	2/5	3/5	3/5	52.93±8.69	50.77±7.46	34.34±4.27	52.05±3.98
PRDC 15 μg (n=5)	300. 05±28.53	2/5	3/5	3/5	3/5	60.17±8.85	57.33±8.12	38.94±2.19	60.73±4.32
**MCT-5W**									
(n=12)	282.36±18.73	8/12	12/12	12/12	4/12	67.35/58.64	64.73/55.4	42.33/36.53	71.44/64.93

All data are showed as Mean ± SD.
